# Cardiometabolic Outcomes Among Adults With Abdominal Obesity and Normal Body Mass Index

**DOI:** 10.1001/jamanetworkopen.2025.37942

**Published:** 2025-10-17

**Authors:** Kedir Y. Ahmed, Setognal B. Aychiluhm, Subash Thapa, Teketo Kassaw Tegegne, Daniel Bekele Ketema, Zemenu Yohannes Kassa, Getiye Dejenu Kibret, Bereket Duko, Desalegn Markos Shifti, Meless G. Bore, Zekariyas Sahile Nezenega, Asres Bedaso, Aklilu Habte Hailegebireal, Habtamu Mellie Bizuayehu, Abel F. Dadi, Tesfalidet Beyene, Mohd. Farooq Shaikh, Tahir A. Hassen, Abdulbasit Seid, Feleke H. Astawesegn, Sewunet Admasu Belachew, Cheru Tesema Leshargie, Fentaw T. Berhe, Utpal K. Mondal, Damien Little, Kasuni Akalanka Hewa Marambage, Shakeel Mahmood, Allen G. Ross

**Affiliations:** 1 Rural Health Research Institute, Charles Sturt University, Orange, New South Wales, Australia; 2 Translational Health Research Institute, Western Sydney University, Campbelltown Campus, New South Wales, Australia; 3 Institute of Public Health, College of Medicine and Health Sciences, University of Gondar, Gondar, Ethiopia; 4 Institute for Physical Activity and Nutrition, Deakin University, Geelong, Victoria, Australia; 5 The George Institute for Global Health, University of New South Wales, Sydney, Australia; 6 School of Public Health, College of Medicine and Health Science, Debre Markos University, Debre Markos, Ethiopia; 7 Center for Health Systems and Safety Research, Australian Institute of Health Innovation, Macquarie University, Sydney, New South Wales, Australia; 8 Research Centre for Public Health, Equity and Human Flourishing, Torrens University Australia, Adelaide, South Australia, Australia; 9 Curtin School of Population Health, Curtin University, Perth, Western Australia, Australia; 10 Child Health Research Centre, The University of Queensland, Brisbane, Australia; 11 The Daffodil Centre, University of Sydney, A Joint Venture With Cancer Council New South Wales, Sydney, Australia; 12 School of Public Health, College of Medicine and Health Sciences, Wachemo University, Hosanna, Ethiopia; 13 Faculty of Health and Environmental Sciences, Auckland University of Technology, Auckland, New Zealand; 14 First Nations Cancer and Wellbeing Research Program, School of Public Health, The University of Queensland, Brisbane, Australia; 15 Menzies School of Health Research, Charles Darwin University, Darwin, Northern Territory, Australia; 16 Addis Continental Institute of Public Health, Addis Ababa, Ethiopia; 17 Centre for Women’s Health Research, School of Medicine and Public Health, College of Health, Medicine and Wellbeing, University of Newcastle, Callaghan, New South Wales, Australia; 18 School of Dentistry and Medical Sciences, Charles Sturt University, Orange, New South Wales, Australia; 19 School of Public Health and Preventive Medicine, Monash University, Melbourne, Victoria, Australia; 20 Department of Epidemiology and Biostatistics, School of Public Health, College of Medicine and Health Sciences, Wollo University, Dessie, Ethiopia; 21 School of Medicine and Dentistry, Griffith University, Gold Coast, Queensland, Australia; 22 Now with College of Medicine, Ajman University, Ajman, United Arab Emirates

## Abstract

**Importance:**

Cardiometabolic disorders are the leading causes of death and disability worldwide, with abdominal obesity being a major contributor to these conditions. Data on normal-weight abdominal obesity and its association with cardiometabolic outcomes are limited.

**Objective:**

To investigate the global prevalence of normal-weight abdominal obesity and its association with cardiometabolic outcomes.

**Design, Setting, and Participants:**

This cross-sectional study used data from the World Health Organization Stepwise Approach to Surveillance of Noncommunicable Disease Risk Factors survey datasets between 2000 and 2020. The surveys were from 91 countries across Africa, the Americas, the Eastern Mediterranean region, Europe, Southeast Asia, and the Western Pacific region. Adults aged 15 to 69 years or 18 to 69 years (based on participating countries’ national definitions of adult) were included. The data were analyzed between April 2024 and January 2025.

**Exposure:**

Normal-weight abdominal obesity, which is defined as a normal body mass index (BMI) of 18.5 to 24.9 (calculated as weight in kilograms divided by height in meters squared) but high waist circumference (female, ≥80 cm; male, ≥94 cm).

**Main Outcomes and Measures:**

The main outcomes were hypertension, diabetes, cholesterol, and triglycerides. Associations with these cardiometabolic outcomes were quantified using multivariable binary logistic regression models.

**Results:**

The study included 471 228 participants (mean [SD] age, 40.4 [15.9] years; 57.8% female). Globally, 21.7% (95% CI, 21.5%-21.8%) of participants with a normal BMI had abdominal obesity, ranging from 15.3% (95% CI, 15.0%-15.7%) in the Western Pacific region to 32.6% (95% CI, 31.9%-33.3%) in the Eastern Mediterranean region. Lebanon had the highest prevalence of normal-weight abdominal obesity (58.4%; 95% CI, 54.1%-62.6%), while Mozambique had the lowest (6.9%; 95% CI, 5.9%-8.1%). Factors associated with abdominal obesity included primary and secondary or higher education (odds ratio [OR], 1.53 [95% CI, 1.50-1.57] and 2.38 [95% CI, 2.33-2.43], respectively), unemployment (OR, 1.25 [95% CI, 1.23-1.27]), low fruits and vegetables intake (OR, 1.22 [95% CI, 1.20-1.24]), and physical inactivity (OR, 1.60 [95% CI, 1.57-1.63]). Additionally, having a normal BMI and abdominal obesity was consistently associated with hypertension (OR, 1.29 [95% CI, 1.25-1.33]), diabetes (OR, 1.81 [95% CI, 1.72-1.90]), high total cholesterol (OR, 1.39 [95% CI, 1.35-1.44]), and high triglycerides (OR, 1.56 [95% CI, 1.48-1.64]).

**Conclusions and Relevance:**

In this cross-sectional study, more than 1 in 5 adults worldwide with a normal BMI had abdominal obesity. Relying solely on BMI may be insufficient to identify these high-risk individuals and provide timely interventions. The findings have implications for the United Nations’ Sustainable Development Goal targets 2.2 (ending all forms of malnutrition) and 3.4 (reducing premature mortality from noncommunicable diseases).

## Key Points

**Question:**

What is the estimated global prevalence of normal-weight abdominal obesity, and is it associated with cardiometabolic outcomes?

**Findings:**

In this cross-sectional study of 471 228 participants, the prevalence of normal-weight abdominal obesity was 21.7%, with the highest in Eastern Mediterranean and lowest in the Western Pacific regions. Normal-weight abdominal obesity was consistently associated with hypertension, diabetes, high total cholesterol, and elevated triglycerides globally.

**Meaning:**

These findings suggest that public health initiatives, such as the promotion of regular physical exercise and a healthy diet, should target not only individuals with overweight or obesity but also those with a normal weight and abdominal obesity.

## Teaser

This cross-sectional study investigates the global prevalence of abdominal obesity with a normal body mass index (BMI) and its association with cardiometabolic outcomes among individuals aged 15 to 69 years.

## Introduction

Cardiometabolic disorders are the leading causes of death and disability globally.^1^ Over the past 3 decades (1990-2019), the global burden of cardiovascular diseases has risen substantially, with cases increasing from 271 million to 523 million and disability-adjusted life-years nearly doubling.^2^ Similarly, diabetes prevalence has surged, with an estimated 828 million adults affected in 2022, an increase of 630 million since 1990.^3^ This growing epidemic is largely driven by environments that promote unhealthy eating and limit physical activity, with adiposity, particularly abdominal obesity, being the most modifiable risk factor contributing to these conditions.^4^ Abdominal obesity (especially excess visceral fat around the abdominal organs) disrupts metabolic processes through inflammatory cytokines and hormones, leading to insulin resistance, dyslipidemia, high blood pressure, and impaired glucose regulation.^4,5,6^

While body mass index (BMI) is commonly used to assess body fat and cardiometabolic risk, it often fails to capture body fat distribution.^7^ Studies showed that individuals with a normal BMI but higher waist circumference face greater cardiovascular risk and mortality, likely due to visceral fat accumulation, which BMI does not adequately measure.^8,9,10^ For example, a study from the US found that half of nonpregnant women with a high body fat percentage were not identified by BMI.^9^ Moreover, individuals with a normal BMI but larger waist circumference are at a higher risk of cardiovascular disorders and mortality than those with a BMI in the overweight or obese range.^10,11^ Despite recommendations to include waist circumference as a vital sign,^12^ global guidelines continue to rely on BMI as the primary measure in routine clinical practice to classify obesity and manage associated cardiometabolic risks.^12^

In the Sustainable Development Goal developed by United Nations member states in 2015, target 3.4 aims to reduce premature mortality from noncommunicable diseases (NCDs), including cardiovascular disease, cancer, diabetes, and chronic respiratory disease, by one-third by 2030.^13^ Addressing these NCDs linked to abdominal obesity will be essential to successfully accomplish this goal. Previously published studies on abdominal obesity and cardiometabolic outcomes are limited to specific countries or regions.^5,8,14,15,16^ To address this research gap, we aimed to answer the following research questions: What is the estimated prevalence of normal-weight abdominal obesity, and is it associated with cardiometabolic outcomes, such as hypertension, diabetes, high total cholesterol, and high triglycerides?

## Methods

### Data Sources

This cross-sectional study used the World Health Organization (WHO) Stepwise Approach to Surveillance of Noncommunicable Disease Risk Factors (STEPs) survey datasets from 91 countries between 2000 and 2020. This study is a secondary analysis of WHO STEPS survey datasets after approval from the WHO NCD Microdata Repository. Ethical clearance was obtained for all surveys from the respective countries. Informed consent for this study was waived as secondary data were used. This study adhered to the Strengthening the Reporting of Observational Studies in Epidemiology (STROBE) reporting guideline for cross-sectional studies.

The WHO initiated the STEPS survey to address the growing need for data on key NCD risk factors.^17^ These risk factors were tobacco use, alcohol use, physical inactivity, and unhealthy diet, as well as key risk factors, including overweight and obesity, raised blood pressure, raised blood glucose, and abnormal blood lipid levels.^17^ The included countries spanned 6 WHO regions: Africa, the Americas, the Eastern Mediterranean, Europe, Southeast Asia, and the Western Pacific. A list of countries and their respective sample sizes is provided in eTable 1 in Supplement 1.

### Design, Sampling, and Population

The WHO STEPS survey used a multistage cluster sampling technique to ensure national representativeness. In each respective country, sample sizes were determined by considering the desired level of the CI, an acceptable margin of error, estimated design effect, projected baseline levels of behaviors being measured, the required number of age-sex estimates, and anticipated nonresponse rate.^18^ This study included adults aged 15 to 69 years who had lived at their current address for at least 6 months (eFigure in Supplement 1). For this study, we included individuals 15 to 69 years as some countries collected data within this range to define adults based on their national standards. Each country followed its own sampling procedure.

### Data Collection

Data for STEPS surveys were collected electronically via personal digital assistants through face-to-face interviews after obtaining informed consent from participants. Surveys were conducted in 3 steps. First, sociodemographic information, behavioral measures (including tobacco and alcohol consumption), physical activity, food hygiene, oral health, cervical cancer screening, and knowledge of NCD risk factors were assessed. Second, physical measurements such as weight, height, waist circumference, and blood pressure were obtained. Third, blood glucose and blood cholesterol levels were measured.

### Cardiometabolic Disorders

The main outcomes were diabetes, hypertension, high total cholesterol, and high triglycerides. Diabetes was defined as having a fasting blood glucose of at least 126 mg/dL (7 mmol/L), use of blood glucose–lowering medication, and/or a self-reported diagnosis.^15^ Hypertension was defined as systolic blood pressure of at least 140 mm Hg, diastolic blood pressure of at least 90 mm Hg, and/or current use of antihypertensive medication.^16^ High cholesterol was defined as total cholesterol of at least 190 mg/dL (5 mmol/L).^19^ High triglycerides were defined as triglycerides of at least 150 mg/dL (1.7 mmol/L).^20^

### Anthropometric Measurements

In the WHO STEPS surveys, anthropometric measurements, including height, weight, and waist circumference, were taken with participants barefoot and wearing light clothing. Height and weight were recorded to the nearest 1 cm and 1 kg, respectively, while waist circumference was measured at the iliac crest and umbilicus to the nearest 1 cm. Body mass index was calculated as weight in kilograms divided by height in meters squared. Abdominal obesity was defined as a waist circumference of at least 80 cm for nonpregnant female participants and at least 94 cm for male participants, following WHO recommendations for identifying increased risk.^14,21^ For waist circumference, a conservative approach was used for this study by using the lower cutoff to ensure worldwide applicability. Body mass index was grouped into 3 categories: normal weight (18.5-24.9), overweight (25.0-29.9), and obese (≥30.0). Normal-weight abdominal obesity refers to individuals within the normal BMI range but with central obesity.

### Other Explanatory Variables

Explanatory variables were broadly grouped as sociodemographic and lifestyle risk factors. Sociodemographic factors included sex (female or male), age (15-29 years, 30-44 years, 45-59 years, or 60-69 years), educational status (no formal schooling, primary education, or secondary or higher education), and occupational status (not working or working). The lifestyle risk factors were daily alcohol intake, current smoking, daily recommended servings of fruits and vegetables, and physical activity.

Physical activity was measured using the WHO Global Physical Activity Questionnaire, which includes work-related, transportation-related, and leisure time physical activity, each grouped as vigorous or moderate intensity.^22^ Frequency, duration, and intensity were coded into metabolic equivalents (METs), for which 1.0 MET equals energy expenditure while sitting quietly for 1 hour. Based on the compendium of physical activities by Ainsworth et al,^23^ vigorous work-related physical activity was assigned 8.0 METs and moderate work-related, transportation-related, and leisure time physical activities were each assigned 4.0 METs. Total weekly METs were calculated by multiplying the duration (minutes) of each activity by its corresponding METs and summing the results. For this study, we grouped the physical activity as physically active (≥600 METs) and physically inactive (<600 METs), consistent with previously published studies and guidelines.^22,24^

### Statistical Analysis

The data were analyzed between April 2024 and January 2025. Data were cleaned using Stata/SE, version 18 (StataCorp LLC) and analyzed using R, version 4.5.1 (R Foundation for Statistical Computing) implemented in the open access Google Colab (Google Research). Biochemical data (eg, cholesterol, triglycerides, fasting blood glucose) were reported in different units across countries, with some using milligrams per deciliter and others millimoles per liter. For comparability, values reported in milligrams per deciliter were converted to millimoles per liter using standard conversion factors. Implausible numeric values (eg, waist circumference >150 cm) were excluded, and categorical variables were recoded for consistency across countries. For transparency, sample Stata code from 1 country and R scripts for the data analysis are included in eAppendixes 1 and 2 in Supplement 1.

Descriptive statistics (frequencies, percentages, means, SDs) were used to characterize participants. Prevalence estimates of abdominal obesity, normal-weight abdominal obesity, and cardiometabolic outcomes were calculated by sociodemographic and lifestyle factors, country, and WHO region (Table; eTable 1 in Supplement 1). Normal-weight abdominal obesity refers to individuals with a normal BMI (18.5-24.9) but high waist circumference (female, ≥80 cm; male, ≥94 cm). Multivariable logistic regression models examined associations between sociodemographic and lifestyle factors and abdominal obesity and between obesity patterns and cardiometabolic outcomes. All models examining the association between obesity patterns and cardiometabolic outcomes were adjusted for age, sex, education, employment, smoking, alcohol use, diet, and physical activity. No multicollinearity was detected (variance inflation factors <5).

**Table.** Prevalence of Cardiometabolic Risk Indicators by World Health Organization Region, 2000 to 2020

Variable	Africa (n = 175 156)		Americas (n = 25 434)		Eastern Mediterranean (n = 52 061)		Europe (n = 38 955)		Southeast Asia (n = 63 044)		Western Pacific (n = 116 578)		Global (N = 471 228)	
	No. of participants	Prevalence, % (95% CI)	No. of participants	Prevalence, % (95% CI)	No. of participants	Prevalence, % (95% CI)	No. of participants	Prevalence, % (95% CI)	No. of participants	Prevalence, % (95% CI)	No. of participants	Prevalence, % (95% CI)	No. of participants	Prevalence, % (95% CI)
**Abdominal obesity**														
Yes	64 629	37.9 (37.7-38.1)	13 016	58.1 (57.4-58.7)	30 133	61.4 (60.9-61.8)	23 027	61.6 (61.1-62.1)	19 347	31.4 (31.1-31.8)	52 401	49.7 (49.4-50.0)	202 553	45.4 (45.2-45.5)
No	105 849	62.1 (61.9-62.3)	9397	41.9 (41.3-42.6)	18 955	38.6 (38.1-39.0)	14 329	38.4 (37.9-38.9)	42 193	68.6 (68.2-68.9)	53 012	50.3 (50.0-50.6)	243 735	54.6 (54.5-54.8)
**Normal-weight obesity**														
Yes	20 565	21.5 (21.3-21.8)	1862	25.8 (24.8-26.8)	5634	32.6 (31.9-33.3)	3640	27.7 (26.9-28.5)	7293	21.1 (20.7-21.5)	6497	15.3 (15.0-15.7)	45 491	21.7 (21.5-21.8)
No	74 937	78.5 (78.2-78.7)	5354	74.2 (73.2-75.2)	11 629	67.4 (66.7-68.1)	9502	72.3 (71.5-73.1)	27 283	78.9 (78.5-79.3)	35 898	84.7 (84.3-85.0)	164 603	78.3 (78.2-78.5)
**Hypertension**														
Yes	41 850	24.2 (24.0-24.4)	7195	28.3 (27.7-28.8)	14 696	28.2 (27.8-28.6)	14 106	36.2 (35.7-36.7)	15 235	24.2 (23.8-24.5)	43 079	36.9 (36.7-37.2)	136 161	29.1 (28.9-29.2)
No	130 757	75.8 (75.6-76.0)	18 239	71.7 (71.1-72.3)	37 365	71.8 (71.4-72.1)	24 849	63.8 (63.3-64.3)	47 809	75.8 (75.5-76.2)	73 499	63.1 (62.8-63.3)	332 518	70.9 (70.8-71.1)
**High total cholesterol**														
Yes	24 843	24.7 (24.4-25.0)	4752	45.1 (44.2-46.7)	11 371	30.1 (29.6-30.5)	7504	26.7 (26.1-27.2)	10 819	30.6 (30.1-31.1)	21 521	28.2 (27.9-28.6)	80 810	28.0 (27.8-28.2)
No	75 736	75.3 (75.0-75.6)	5775	54.9 (53.9-55.8)	26 436	69.9 (69.5-70.4)	20 641	73.3 (72.8-72.8)	24 558	69.4 (68.9-69.9)	54 682	71.8 (71.4-72.1)	207 828	72.0 (71.8-72.2)
**High triglycerides**														
Yes	8743	31.2 (30.6-31.7)	921	25.5 (24.1-26.9)	7424	27.3 (26.7-27.8)	3376	34.5 (33.6-35.4)	6219	33.7 (33.0-34.4)	5952	28.5 (27.9-29.1)	32 635	30.2 (29.9-30.5)
**No**	19 286	68.8 (68.3-69.3)	2692	74.5 (73.1-75.9)	19 804	72.7 (72.2-73.3)	6413	65.5 (64.6-66.4)	12 241	66.3 (65.6-67.0)	14 926	71.5 (70.9-72.1)	75 362	69.8 (69.5-70.0)
**Diabetes**														
Yes	10 073	8.2 (8.0-8.4)	2057	19.6 (18.9-20.4)	5176	13.9 (13.6-14.3)	2923	8.6 (8.3-8.9)	3316	9.5 (9.2-9.8)	9319	11.8 (11.6-12.0)	32 864	10.3 (10.2-10.4)
No	112 671	91.8 (91.6-91.9)	8423	80.4 (79.6-81.1)	31 961	86.1 (85.7-86.4)	30 912	91.4 (91.0-91.7)	31 671	90.5 (90.2-90.8)	69 649	88.2 (88.0-88.4)	285 287	89.7 (89.6-89.8)

^a^ Abdominal obesity was considered as a waist circumference of 80 cm or higher for female individuals and 94 cm or higher for male individuals.

^b^ Normal weight was considered a body mass index (calculated as weight in kilograms divided by height in meters squared) of 18.5 to 24.9 (vs 25.0-29.9 for overweight and ≥30.0 for obesity).

E-values were computed to assess the potential impact of unmeasured confounding in the associations between obesity and cardiometabolic outcomes (eTables 4 and 5 in Supplement 1). Interaction terms were tested for sex, age, and education level in regression models (eTable 6 in Supplement 1). All analyses were stratified by the 6 WHO regions, and results are reported as odds ratios (ORs) with 95% CIs. Significance was considered when the 95% CI did not include 1.00.

## Results

### Study Participants

This study included 471 228 participants aged 15 to 69 years (mean [SD] age, 40.4 [15.9] years; 57.8% female and 42.2% male; 37.2% from Africa, 5.4% from the Americas, 11.0% from the Eastern Mediterranean region, 8.3% from Europe, 13.4% from Southeast Asia, and 24.7% from the Western Pacific region) (eTable 2 in Supplement 1). Overall, 43.6% had attained a secondary level of education or higher, ranging from 30.8% in Africa to 90.2% in Europe. Of the participants, 36.3% were aged 15 to 29 years, and 37.8% were not working. Globally, 6.2% participants had 10 or more standard alcohol drinks in the past week, and 19.1% were current smokers. Daily vegetable consumption was reported by 45.3% of participants, but only 19.6% consumed fruits daily. Additionally, 28.5% of participants were physically inactive.

### Prevalence of Abdominal Obesity

The global prevalence of abdominal obesity was 45.4%, ranging from 31.4% (95% CI, 31.1%-31.8%) in Southeast Asia to 61.6% (95% CI, 61.1%-62.1%) in Europe (Table). Country-level prevalence ranged from 89.2% (95% CI, 88.4%-89.9%) in Tonga to 12.7% (95% CI, 12.2%-13.2%) in Vietnam (eTable 1 in Supplement 1). Among participants with normal BMI, 21.7% (95% CI, 21.5%-21.8%) had abdominal obesity, ranging from 15.3% (95% CI, 15.0%-15.7%) in the Western Pacific region to 32.6% (95% CI, 31.9%-33.3%) in the Eastern Mediterranean region (Table). Lebanon (58.4% [95% CI, 54.1%-62.6%]) and Morocco (45.7% [95% CI, 43.4%-47.9%]) had the highest country-level prevalence, while Mozambique had the lowest (6.9% [95% CI, 5.9%-8.1%]) (eTable 1 in Supplement 1).

### Risk Factors Associated With Abdominal Obesity

Globally, a primary or secondary or higher education level was associated with abdominal obesity (OR, 1.53 [95% CI, 1.50-1.57] and 2.38 [95% CI, 2.33-2.43], respectively) compared with no formal schooling. However, in Africa, a secondary level of education or higher was associated with a lower odds of abdominal obesity (OR, 0.64 [95% CI, 0.56-0.73]). Being unemployed was associated with a higher odds of abdominal obesity in Africa (OR, 1.20 [95% CI, 1.11-1.30]), Southeast Asia (OR, 1.36 [95% CI, 1.31-1.41]), and the Western Pacific region (OR, 1.42 [95% CI, 1.34-1.50]) but a lower odds in the Americas (OR, 0.88 [95% CI, 0.82-0.93]). Consumption of less than 5 servings of fruits and vegetables per day was associated with a higher odds of abdominal obesity (OR, 1.22 [95% CI, 1.20-1.24]) but with a lower odds in Africa (OR, 0.88 [95% CI, 0.83-0.94]). Physical inactivity was associated with an increased risk of abdominal obesity across all regions (OR, 1.60 [95% CI, 1.57-1.63]) (eTable 3 in Supplement 1).

### Abdominal Obesity and Cardiometabolic Outcomes

**Figure 1.**
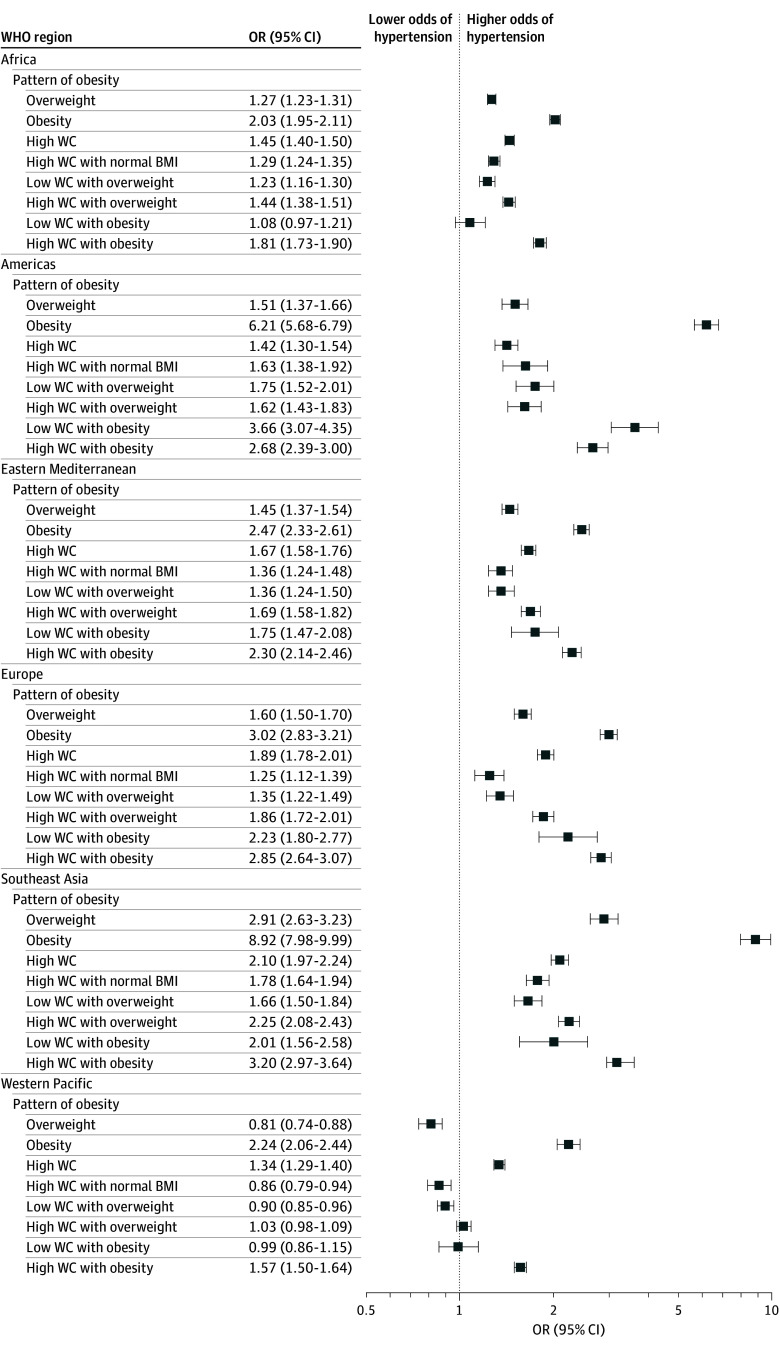
Associations Between Different Patterns of Obesity and Hypertension Across 6 World Health Organization (WHO) Regions, 2000 to 2020 All associations between obesity patterns and hypertension were adjusted for age, sex, educational status, occupational status, smoking, alcohol use, fruits and vegetables consumption, and physical activity. Reference categories were normal body mass index (BMI, calculated as weight in kilograms divided by height in meters squared), low waist circumference (WC), and low WC and normal BMI. Body mass index categories were 18.5 to 24.9 for normal weight, 25.0 to 29.9 for overweight, and 30.0 or higher for obesity. Abdominal obesity was considered as a waist circumference of 80 cm or higher for female individuals and 94 cm or higher for male individuals.

**Figure 2.**
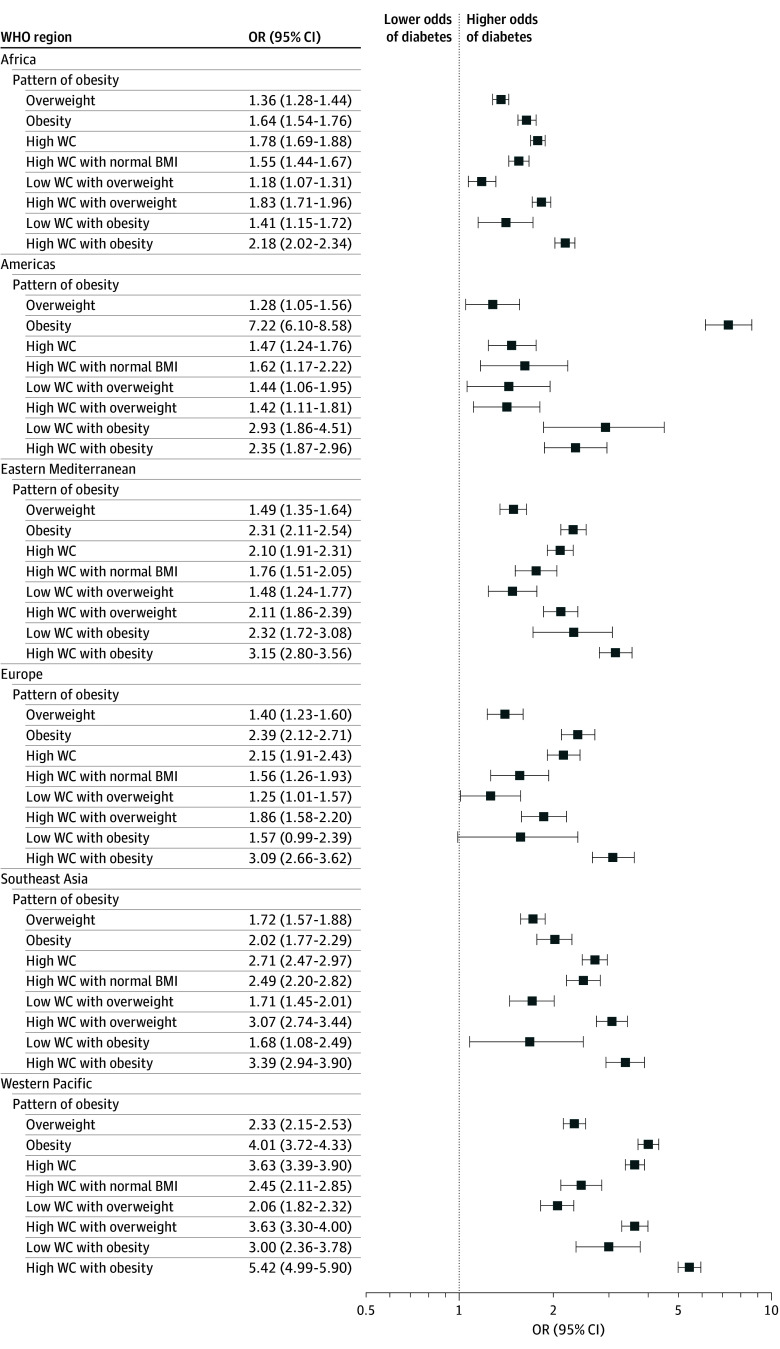
Associations Between Different Patterns of Obesity and Diabetes Across World Health Organization (WHO) Regions, 2000 to 2020 All associations between obesity patterns and diabetes were adjusted for age, sex, educational status, occupational status, smoking, alcohol use, fruits and vegetables consumption, and physical activity. Reference categories were normal body mass index (BMI, calculated as weight in kilograms divided by height in meters squared), low waist circumference (WC), and low WC and normal BMI. Body mass index categories were 18.5 to 24.9 for normal weight, 25.0 to 29.9 for overweight, and 30.0 or higher for obesity. Abdominal obesity was considered as a waist circumference of 80 cm or higher for female individuals and 94 cm or higher for male individuals.

Abdominal obesity was associated with a higher odds of hypertension across all regions compared with no abdominal obesity (OR, 1.58 [95% CI, 1.55-1.61]). Abdominal obesity with a normal BMI was also associated with a higher odds of hypertension globally (OR, 1.29 [95% CI, 1.25-1.33]), except in the Western Pacific region (Figure 1; eTable 4 in Supplement 1). Additionally, abdominal obesity was associated with diabetes across all regions (OR, 2.30 [95% CI, 2.23-2.37]), as was abdominal obesity with a normal BMI (OR, 1.81 [95% CI, 1.72-1.90]) (Figure 2; eTable 4 in Supplement 1).

**Figure 3.**
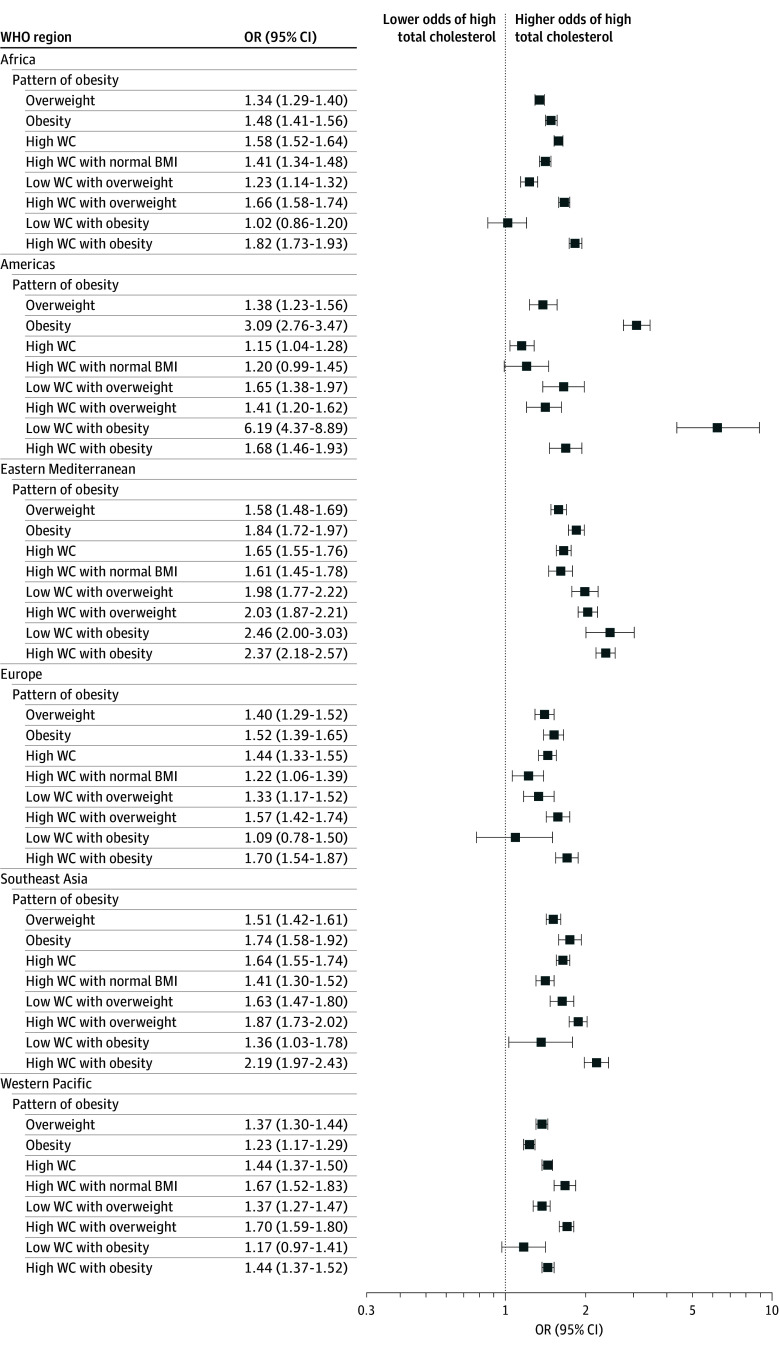
Associations Between Different Patterns of Obesity and High Total Cholesterol Across World Health Organization (WHO) Regions, 2000 to 2020 All associations between obesity patterns and total cholesterol were adjusted for age, sex, educational status, occupational status, smoking, alcohol use, fruits and vegetables consumption, and physical activity. Reference categories were normal body mass index (BMI, calculated as weight in kilograms divided by height in meters squared), low waist circumference (WC), and low WC and normal BMI. Body mass index categories were 18.5 to 24.9 for normal weight, 25.0 to 29.9 for overweight, and 30.0 or higher for obesity. Abdominal obesity was considered as a waist circumference of 80 cm or higher for female individuals and 94 cm or higher for male individuals.

**Figure 4.**
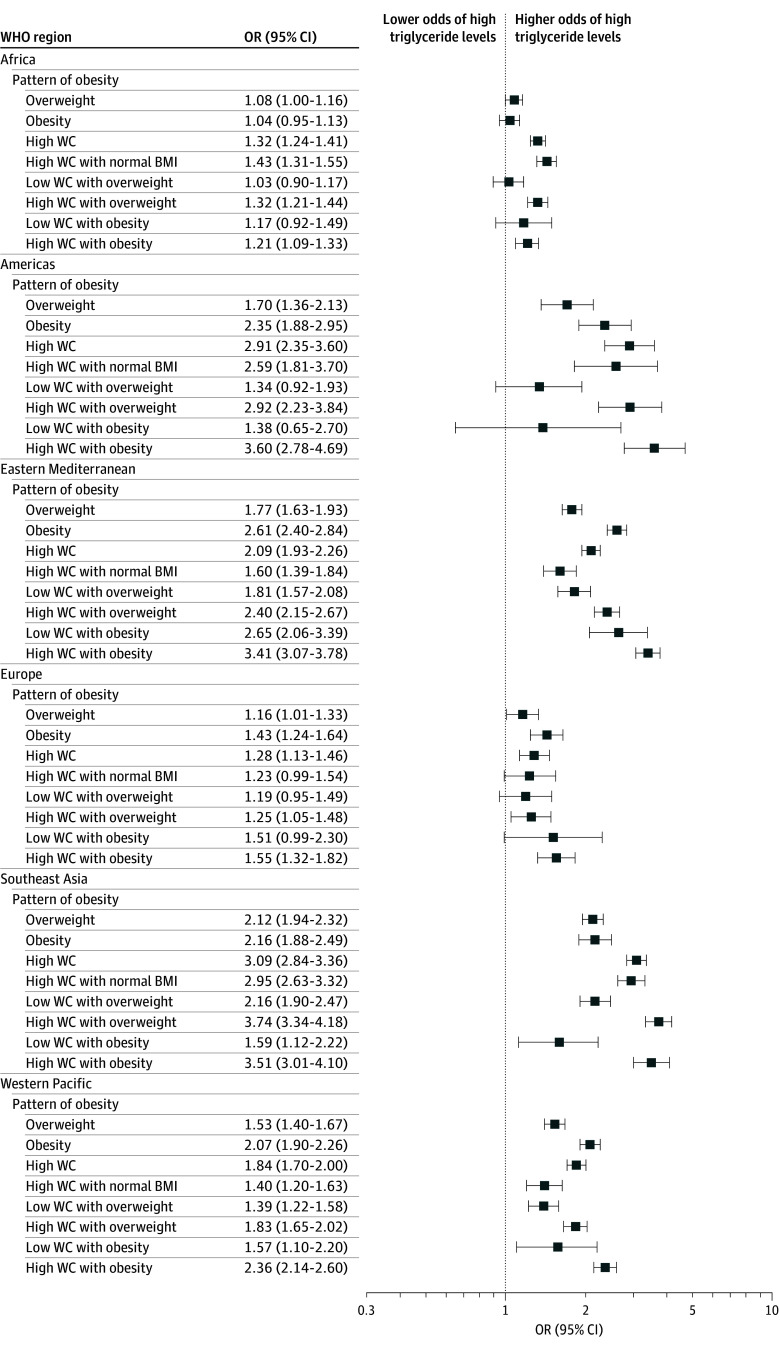
Associations Between Different Patterns of Obesity and High Triglycerides Across World Health Organization (WHO) Regions, 2000 to 2020 All associations between obesity patterns and triglycerides were adjusted for age, sex, educational status, occupational status, smoking, alcohol use, fruits and vegetables consumption, and physical activity. Reference categories were normal body mass index (BMI, calculated as weight in kilograms divided by height in meters squared), low waist circumference (WC), and low WC and normal BMI. Body mass index categories were 18.5 to 24.9 for normal weight, 25.0 to 29.9 for overweight, and 30.0 or higher for obesity. Abdominal obesity was considered as a waist circumference of 80 cm or higher for female individuals and 94 cm or higher for male individuals.

Abdominal obesity was associated with elevated total cholesterol across all regions (OR, 1.49 [95% CI, 1.46-1.53]). Abdominal obesity with a normal BMI also was associated with elevated total cholesterol globally (OR, 1.39 [95% CI, 1.35-1.44]), except in the Americas (Figure 3; eTable 5 in Supplement 1). Additionally, abdominal obesity was associated with elevated triglycerides across all regions (OR, 1.60 [95% CI, 1.55-1.66]), as was abdominal obesity with a normal BMI (OR, 1.56 [95% CI, 1.48-1.64]), except in Europe (Figure 4; eTable 5 in Supplement 1).

### Interactions on the Associations Between Abdominal Obesity and Cardiometabolic Conditions

Interaction analyses by sex, age, and education level revealed a higher odds of hypertension with abdominal obesity among female participants (OR, 1.66 [95% CI, 1.62-1.71]) compared with male participants (OR, 1.55 [95% CI, 1.51-1.60]), while male participants with abdominal obesity had a higher odds of elevated triglycerides (OR, 1.85 [95% CI, 1.76-1.95]) compared with female participants (OR, 1.44 [95% CI, 1.38-1.51]), with regional differences noted. The highest odds of diabetes and hypertension were observed among older age groups and of all outcomes assessed among participants with primary or lower education. Full interaction results are provided in eTable 6 in Supplement 1.

## Discussion

This cross-sectional study is, to our knowledge, the first global examination of the association between normal-weight (and normal BMI) abdominal obesity and cardiometabolic outcomes using data from a large number of population-based surveys across multiple countries. This study showed that more than 1 in 5 adults with a normal BMI had abdominal obesity, with the highest prevalence observed in the Eastern Mediterranean region and the lowest in the Western Pacific region. This study also showed that abdominal obesity was associated with risk factors such as female sex, higher education, unemployment, low fruits and vegetables consumption, and physical inactivity, with some variations observed across the world regions. Importantly, normal-weight abdominal obesity was consistently associated with cardiometabolic conditions, such as hypertension, diabetes, high total cholesterol, and elevated triglycerides.

While previous studies on abdominal obesity have mostly been region specific or limited to individual outcomes (eg, hypertension),^15,16,25,26^ our multicountry analysis provides a more comprehensive global perspective, exploring associations across 4 key cardiometabolic outcomes (ie, hypertension, diabetes, cholesterol, triglycerides). The importance of fat topography over general adiposity was first emphasized by Jean Vague,^27^ who distinguished between apple-shaped and pear-shaped obesity. Since, evidence has shown that abdominal (visceral) fat provides a stronger estimate of cardiometabolic risk than BMI alone due to its inflammatory and metabolic effects.^4,5,10,16,28^ As such, expert groups, including the International Atherosclerosis Society and International Chair on Cardiometabolic Risk, have recommended routine waist circumference measurement in clinical settings.^12^ Nevertheless, global guidelines continue to prioritize BMI, potentially overlooking individuals at high risk of preventable disease. Our findings suggest the need to use both BMI and waist circumference together, rather than in isolation, to provide a more complete and accurate assessment of cardiometabolic risk across diverse populations.

Our findings also underscore regional and socioeconomic differences. Abdominal obesity was more prevalent in wealthier regions, such as Europe, the Americas, the Western Pacific, and the Eastern Mediterranean, while lower rates were found in Africa and Southeast Asia, consistent with previous reviews.^29^ However, it is important to note that low- and middle-income countries are also increasingly vulnerable to rising obesity rates due to rapid urbanization, internal migration, dietary shifts, and physical inactivity.^30,31^ This vulnerability is supported by our results showing that higher education is associated with lower abdominal obesity risk in Africa, which may be due to improved health literacy, while employment has reduced the risk, possibly due to the physical demands of certain jobs and limited access to processed foods. Traditional diets rich in whole grains, legumes, and vegetables have contributed to lower obesity rates in these regions, but these practices are eroding due to urbanization and changing food environments, especially among wealthier populations.^32^

Obesity is a complex, multifactorial health issue arising from the interplay of genetic, environmental, dietary, and lifestyle factors. Despite worldwide efforts to improve health behaviors and extensive research into its pathophysiology, obesity has continued to rise, reaching epidemic levels globally. This rise has substantially contributed to the burden of chronic conditions, such as cardiovascular disease, type 2 diabetes, and certain types of cancers. Addressing this epidemic requires commitments from global health agencies and national governments, along with substantial funding for interventions that combine individual- and population-level approaches to address root causes, including socioeconomic disparities, cultural norms, chronic psychological stress, and limited access to healthy food and physically active environments.

Several countries have implemented population-level policies to combat obesity, such as sugar taxes, improved access to healthy foods, physical activity incentives, and metabolic screening.^33,34,35^ Japan’s Metabo-Law requires adults to undergo annual waistline measurements during health checkups. Patients at risk for central obesity and cardiometabolic diseases are referred to counseling and provided with ongoing motivational support. While Japan’s policies are considered paternalistic, strong regulations can be justified given disparities in sociocultural norms around healthy weight, which contribute to varying obesity and cardiometabolic risk levels.^34^

Similar to global tobacco control successes, strong regulations, public campaigns, and legislative action could be effective. However, softer approaches emphasizing individual responsibility have also shown success.^35^ For example, in the early 2000s, Iceland introduced policies promoting healthy food marketing, school-based campaigns, and increased access to sports activities, particularly targeting youths.^36^ South Korea’s promotion of traditional diets (eg, the K-diet) has also contributed to its low rates of abdominal obesity and cardiometabolic risks.^37^ In contrast, many low- and middle-income countries and Eastern Mediterranean countries have yet to adopt comprehensive, long-term measures for obesity prevention at the population level.

### Strengths and Limitations

The main strengths of our study were its global data and broad scope, which enabled regional comparisons. With a large number of participants, we were able to robustly assess the associations between BMI and waist circumference, both within and across regions, with hypertension, diabetes, total cholesterol, and triglycerides.

Our study also had several limitations. First, our results were based on cross-sectional data, which presented challenges in establishing a temporal association between modifiable risk factors and health outcomes, although our findings align with those of previously published longitudinal studies.^5,38^ Second, there are limitations in data pooling and analyses, especially when data are collected across different countries and periods. Third, many highly developed countries do not conduct the WHO STEPS survey, which prevented us from including data from these regions and may have influenced the representativeness of our findings. Fourth, we were unable to account for all potential confounders, such as health service factors and social participation, which may have influenced the observed associations. To assess the robustness of our findings to unmeasured confounding, we calculated E-values. Given that the E-values were relatively small, even modest unmeasured confounding could potentially influence the observed associations, and this should be considered when interpreting our results (eTables 4 and 5 in Supplement 1). Fifth, we lacked data on direct measures of body composition or fat distribution (eg, computed tomography scans, magnetic resonance imaging) as these measurements are more complex and not feasible in large-scale population surveillance, particularly in settings with limited health care resources. Finally, most behavioral factors (eg, physical activity, dietary intake, alcohol consumption) were self-reported and, thus, subject to potential recall and social desirability biases. Nonetheless, the WHO STEPS methodology has been extensively validated across diverse populations.^17^

## Conclusions

This cross-sectional study showed that more than 1 in 5 adults with a normal BMI globally have abdominal obesity. Normal-weight abdominal obesity was consistently associated with hypertension, diabetes, high total cholesterol, and high triglycerides, with minor regional differences observed. Relying solely on BMI may not be sufficient for identifying high-risk individuals and ensuring timely interventions. Additionally, addressing the global obesity epidemic requires an ecologic approach that integrates both individual- and population-based interventions while tackling societal root causes, such as socioeconomic disparities, chronic stress, and limited access to healthy food and physically active environments. The findings have implications for the United Nations’s Sustainable Development Goal targets 2.2 (ending all forms of malnutrition) and 3.4 (reducing premature mortality from NCDs).

## Notes

Supplement 1. **eFigure.** Flowchart Showing the Results of Data Cleaning **eTable 1.** List of Countries, Sample Size, and Prevalence of Abdominal Obesity and Normal-Weight Abdominal Obesity **eTable 2.** Study Participant Sociodemographic Characteristics, 2000-2020 **eTable 3.** Risk Factors Associated With Abdominal Obesity by Geographic Region **eTable 4.** Associations Between Different Patterns of Obesity and Diabetes and Hypertension Across 6 WHO Regions Globally From 2000 to 2020 **eTable 5.** Associations Between Different Patterns of Obesity and High Total Cholesterol and Triglycerides Across 6 WHO Regions Globally From 2000 to 2020 **eTable 6.** Interaction of Sex, Age, and Educational Status With the Associations Between Abdominal Obesity and Cardiometabolic Conditions, 2000 to 2020 **eAppendix 1.** Stata Codes for Data Cleaning **eAppendix 2.** R Codes for Regression Models

Supplement 2.
Data Sharing Statement
