# Supplementary material for: Cardiometabolic Outcomes Among Adults With Abdominal Obesity and Normal Body Mass Index
Source: JAMA Netw Open. 2025 Oct 17;8(10):e2537942. doi: 10.1001/jamanetworkopen.2025.37942 (PMC12534855; doi:10.1001/jamanetworkopen.2025.37942)
Supplement: Supplement 1. — eFigure. Flowchart Showing the Results of Data Cleaning eTable 1. List of Countries, Sample Size, and Prevalence of Abdominal Obesity and Normal-Weight Abdominal Obesity eTable 2. Study Participant Sociodemographic Characteristics, 2000-2020 eTable 3. Risk Factors Associated With Abdominal Obesity by Geographic Region eTable 4. Associations Between Different Patterns of Obesity and Diabetes and Hypertension Across 6 WHO Regions Globally From 2000 to 2020 eTable 5. Associations Between Different Patterns of Obesity and High Total Cholesterol and Triglycerides Across 6 WHO Regions Globally From 2000 to 2020 eTable 6. Interaction of Sex, Age, and Educational Status With the Associations Between Abdominal Obesity and Cardiometabolic Conditions, 2000 to 2020 eAppendix 1. Stata Codes for Data Cleaning eAppendix 2. R Codes for Regression Models [file jamanetwopen-e2537942-s001.pdf]

Supplemental Online Content

Ahmed KY, Aychiluhm SB, Thapa S, et al. Cardiometabolic outcomes among adutls with abdominal obesity and normal body mass index. *JAMA Netw Open*. 2025;8(10):e2537942. doi:10.1001/jamanetworkopen.2025.37942

eFigure. Flowchart Showing the Results of Data Cleaning

eTable 1. List of Countries, Sample Size, and Prevalence of Abdominal Obesity and Normal-Weight Abdominal Obesity

eTable 2. Study Participant Sociodemographic Characteristics, 2000-2020

eTable 3. Risk Factors Associated With Abdominal Obesity by Geographic Region

eTable 4. Associations Between Different Patterns of Obesity and Diabetes and Hypertension Across 6 WHO Regions Globally From 2000 to 2020

eTable 5. Associations Between Different Patterns of Obesity and High Total Cholesterol and Triglycerides Across 6 WHO Regions Globally From 2000 to 2020

eTable 6. Interaction of Sex, Age, and Educational Status With the Associations Between Abdominal Obesity and Cardiometabolic Conditions, 2000 to 2020

eAppendix 1. Stata Codes for Data Cleaning

eAppendix 2. R Codes for Regression Models

This supplemental material has been provided by the authors to give readers additional information about their work.

© 2025 Ahmed KY et al. *JAMA Network Open*.

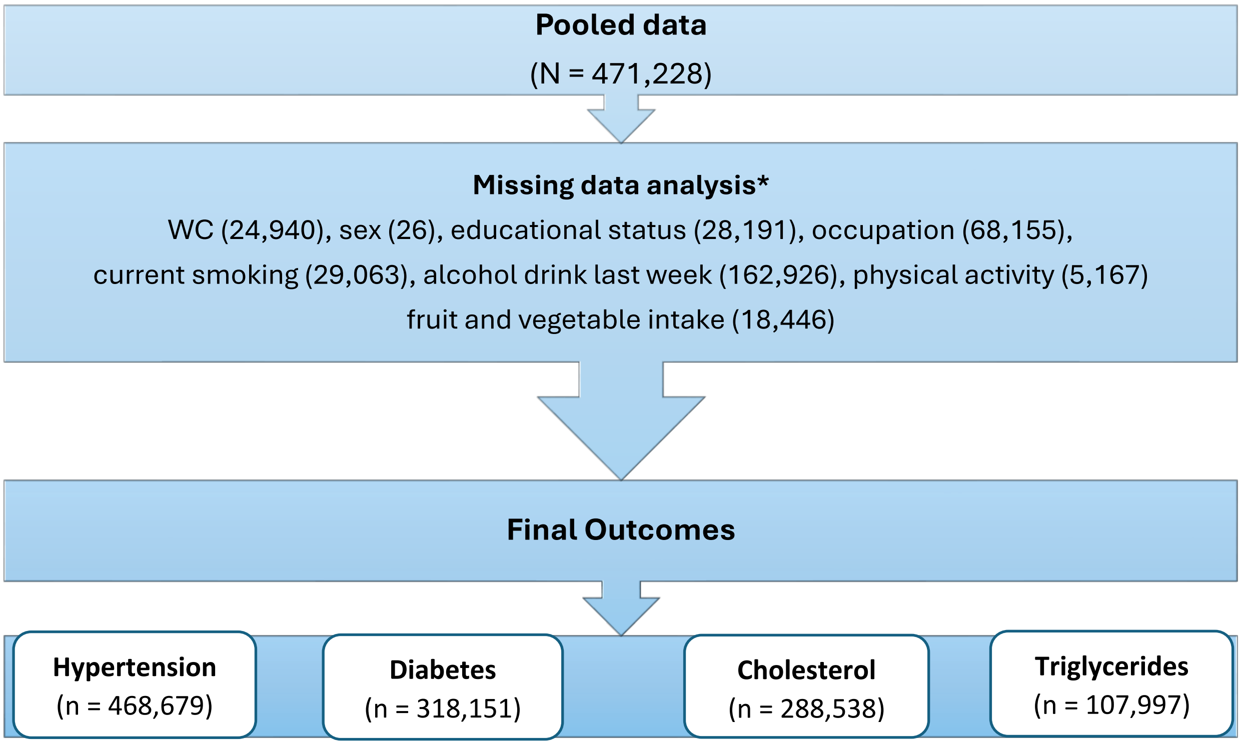

eFigure. Flowchart Showing the Results of Data Cleaning. NB: The number on the brackets of each covariates are the number of missings for each variable.

© 2025 Ahmed KY et al. *JAMA Network Open*.

eTable 1. List of Countries, Sample Size, and Prevalence of Abdominal Obesity and Normal-Weight Abdominal Obesity, 2000-2020

Countries	Sample size	Abdominal obesity with 95% CI			Normal-weight abdominal obesity with 95% CI		
		Prevalence	Lower interval	Upper interval	Prevalence	Lower interval	Upper interval
Afghanistan	3777	50.24	48.64	51.84	27.28	25.26	29.40
Algeria	10884	60.29	59.35	61.21	32.19	30.81	33.59
America Samoa	2013	87.63	86.19	89.07	24.79	22.90	26.68
Armenia	2318	68.07	65.99	70.09	40.75	37.34	44.25
Azerbajan	2778	67.11	65.32	68.84	31.39	28.43	34.51
Bahamas	1643	47.77	45.26	50.30	22.74	18.34	27.84
Bangladesh	17209	25.61	24.96	26.26	18.99	18.23	19.78
Barbados	1281	62.31	57.02	67.33	29.13	21.18	38.60
Belarus	4986	59.87	58.49	61.22	19.64	17.79	21.62
Benin	14188	36.86	36.07	37.66	20.04	19.19	20.91
Bolivia	4383	64.23	62.75	65.68	27.97	25.64	30.42
Botswana	7901	48.89	47.77	50.00	28.19	26.74	29.70
Brunei	3791	53.96	51.69	56.21	14.79	12.10	17.95
Central Africa Republic	7181	39.48	38.35	40.62	29.93	28.53	31.36
Cameroon	9590	37.48	36.50	38.48	11.65	10.76	12.60
Campbodia	5346	22.48	21.37	23.64	13.63	12.55	14.79
Capo Verde	6230	45.15	43.91	46.39	18.66	17.31	20.08
Cayman Islands	1289	58.62	55.85	61.33	14.63	11.23	18.83
Chad	1941	52.61	50.09	55.11	40.87	37.42	44.42
Comoros	5225	55.29	53.92	56.65	32.84	31.01	34.71
Republic of the Congo	1978	37.10	34.99	39.27	22.78	20.49	25.26
Democratic Republic of the Congo	1916	25.07	23.14	27.11	17.03	14.91	19.39
Cook Islands	3267	84.07	82.66	85.38	24.31	19.70	29.59
Cote Divoire	4628	32.73	31.40	34.10	19.42	18.00	20.92
Ecuador	4576	63.04	61.61	64.44	24.83	22.70	27.09
Eritrea	8287	22.72	21.82	23.64	22.25	21.03	23.52
Eswatini	4722	48.44	46.96	49.93	19.44	17.70	21.32
Ethiopia	9431	20.11	19.30	20.93	17.29	16.37	18.26
Fiji	2566	67.19	65.34	68.99	26.86	23.70	30.26
French Polynesia	3469	68.66	67.08	70.21	28.78	26.03	31.69
Gabon	2601	34.74	32.92	36.61	16.18	14.20	18.38
Gambia	3871	27.66	26.19	29.18	21.14	19.32	23.08
Georgia	10587	65.34	64.40	66.27	28.30	26.75	29.91
Ghana	2597	63.44	61.56	65.27	34.23	31.25	37.35

© 2025 Ahmed KY et al. *JAMA Network Open*.

Grenada	1116	57.79	54.82	60.71	25.32	21.24	29.89
Guinea	2386	34.21	32.34	36.14	23.42	21.33	25.65
Guyana	2652	64.87	63.03	66.67	38.72	35.69	41.83
Iraq	3947	77.34	76.00	78.62	39.54	36.42	42.75
Jordan	5527	64.05	62.71	65.37	18.73	16.79	20.83
Kiribati	3870	64.19	62.45	65.89	17.42	14.76	20.45
Kuwait	6080	67.20	65.95	68.42	22.03	19.66	24.59
Kyrgystan	2550	63.09	61.20	64.94	24.32	21.61	27.26
Laos	6669	29.30	28.02	30.61	17.05	15.76	18.42
Lebanon	1886	74.09	71.95	76.13	58.38	54.09	62.55
Lesotho	2269	49.08	46.97	51.18	19.68	17.32	22.27
Liberia	2389	38.70	36.71	40.72	26.18	23.54	29.01
Malawi	8979	28.78	27.84	29.74	16.74	15.82	17.71
Maldives	1737	43.51	41.17	45.88	17.77	15.10	20.78
Mali	4650	46.23	44.79	47.69	29.11	27.32	30.97
Marshal Islands	2944	58.98	56.79	61.14	15.88	13.24	18.93
Mauritania	2549	47.34	45.38	49.32	27.86	25.27	30.61
Micronesia	10316	68.40	67.44	69.35	21.64	20.00	23.36
Moldova	4752	57.91	56.44	59.36	20.09	18.14	22.20
Mongolia	21180	48.20	47.53	48.88	17.84	17.10	18.60
Morocco	5307	71.50	70.27	72.71	45.65	43.43	47.88
Mozambique	3197	20.00	18.59	21.49	6.91	5.88	8.11
Myanmar	8191	31.69	30.67	32.72	17.82	16.71	18.98
Namibia	3189	39.89	38.20	41.60	18.69	16.82	20.73
Nauru	3595	77.46	75.99	78.87	12.99	9.87	16.92
Nepal	9638	29.74	28.83	30.66	21.19	20.18	22.24
Niger	2670	30.94	29.21	32.72	28.73	26.69	30.86
Niue	901	77.29	74.41	79.93	20.00	13.78	28.11
Pakistan	7009	43.45	42.25	44.66	28.53	26.96	30.15
Palau	2187	71.74	69.81	73.60	28.46	24.64	32.60
Palistine	6718	67.66	66.46	68.85	30.29	28.17	32.49
Qatar	2431	71.53	69.66	73.32	26.44	22.88	30.34
Rwanda	7002	23.00	22.02	24.01	15.25	14.29	16.25
Saint Luci	2946	55.46	53.62	57.28	16.67	14.34	19.29
Samoa	4273	74.29	72.92	75.62	14.97	12.21	18.23
São Tomé and Príncipe	4721	42.35	40.91	43.80	22.76	21.14	24.47
Seychelles	1255	60.96	58.23	63.62	18.85	15.39	22.89
Sierraleone	4705	38.77	37.36	40.20	29.60	27.96	31.30
Solomon Islands	5276	48.22	46.71	49.73	13.61	12.04	15.35

© 2025 Ahmed KY et al. *JAMA Network Open*.

Sri Lanka	23714	36.54	35.91	37.17	24.20	23.44	24.97
Sudan	7295	48.15	47.00	49.30	35.40	33.86	36.97
Syria	2083	67.00	64.86	69.06	42.28	38.53	46.13
Tanzania	8033	39.22	38.15	40.30	24.57	23.36	25.81
Tajikistan	2669	47.57	45.67	49.47	22.24	19.91	24.77
Timor-Leste	2555	22.67	21.06	24.36	20.68	18.79	22.71
Togo	4221	24.85	23.56	26.19	15.77	14.50	17.13
Tokelau	1127	78.49	75.97	80.82	20.16	14.00	28.14
Tonga	7133	89.19	88.43	89.91	32.62	28.71	36.79
Turkmenistan	3959	63.33	61.82	64.82	41.74	39.44	44.08
Tuvalu	1144	78.35	75.76	80.73	24.00	17.32	32.25
Uganda	3781	28.60	27.16	30.09	17.58	16.15	19.12
Ukraine	4356	58.56	57.04	60.05	23.72	21.60	25.98
Uruguay	4450	64.06	62.36	65.73	30.48	27.89	33.19
Vanuatu	5985	39.90	38.65	41.16	19.74	18.30	21.28
Vietnam	18276	12.65	12.17	13.15	8.35	7.88	8.84
Wallis Futuna	1250	85.46	83.30	87.39	19.82	13.42	28.27
Zambia	5990	36.98	35.75	38.22	19.30	18.05	20.61

© 2025 Ahmed KY et al. *JAMA Network Open*.

eTable 2. Study Participant Sociodemographic Characteristics, 2000-2020 [N = 471,228]

Variables	Africa (N = 175,156)	Americas [N = 25,434]	Eastern Mediterranean [N = 52,061]	Europe [N = 38,955]	South-East Asian Region [N = 63,044]	Western Pacific [N = 116,578]	Global [N = 471,228]
Gender							
Males	72751 (41.53)	10507 (41.31)	21291 (40.9)	14356 (36.85)	28695 (45.52)	51308 (44.02)	198908 (42.2)
Females	102405 (58.47)	14927 (58.69)	30766 (59.1)	24599 (63.15)	34349 (54.48)	65248 (55.98)	272294 (57.8)
Age group							
15-29 years	51027 (29.13)	5127 (20.16)	14727 (28.29)	6682 (17.15)	13371 (21.21)	26642 (22.85)	171098 (36.3)
30-44 years	65979 (37.67)	8856 (34.82)	18598 (35.72)	11240 (28.85)	23213 (36.82)	43212 (37.07)	133317 (28.3)
45-59 years	42335 (24.17)	7982 (31.38)	13142 (25.24)	13706 (35.18)	19052 (30.22)	37100 (31.82)	117576 (24.9)
60+ years	15817 (9.03)	3469 (13.64)	5594 (10.75)	7327 (18.81)	7408 (11.75)	9624 (8.26)	49237 (10.6)
Educational status							
No formal schooling	48027 (28.45)	1496 (6.17)	10749 (23.06)	132 (0.42)	11025 (18.34)	13548 (12.15)	84977 (19.2)
Primary or less schooling	68869 (40.8)	10234 (42.21)	17622 (37.8)	2993 (9.42)	29258 (48.67)	35746 (32.06)	164722 (37.2)
Secondary or higher	51913 (30.75)	12515 (51.62)	18252 (39.15)	28637 (90.16)	19833 (32.99)	62190 (55.78)	193338 (43.6)
Occupational status							
Not working	54657 (34.66)	5449 (22.4)	27950 (54.07)	18339 (53.21)	12994 (36.87)	32996 (33.11)	152385 (37.8)
Working	103035 (65.34)	18881 (77.6)	23740 (45.93)	16128 (46.79)	22247 (63.13)	66657 (66.89)	250688 (62.2)
Current smoking							
No	147139 (88.95)	20440 (83.27)	41463 (82.82)	31429 (80.85)	12418 (19.71)	66835 (66.68)	357906 (80.9)
Yes	18287 (11.05)	4108 (16.73)	8600 (17.18)	7445 (19.15)	50600 (80.29)	33401 (33.32)	84259 (19.1)
Standard alcohol drinks last week							
Less than 10 drinks	125524 (93.26)	16380 (92.38)	23992 (99.81)	20750 (93.88)	30129 (96.89%)	72530 (92.12)	289305 (93.8)
10 or more drinks	9075 (6.74)	1351 (7.62)	45 (0.19)	1353 (6.12)	966 (3.11%)	6207 (7.88)	18997 (6.2)
Daily fruit consumption							

© 2025 Ahmed KY et al. *JAMA Network Open*.

No	22097 (14.19%)	15298 (67.36%)	40165 (78.83%)	19196 (49.8%)	54411 (88.28)	94230 (82.2%	356876 (80.4)
Yes	22097 (14.19%)	7414 (32.64%)	10787 (21.17%)	19348 (50.2%)	7221 (11.72)	20412 (17.8%)	87279 (19.6)
Daily vegetable consumption							
No	110884 (67.95)	13299 (58.41)	27600 (53.76)	13662 (35.47)	19989 (31.86%)	62401 (54.27)	247835 (54.7)
Yes	52308 (32.05)	9468 (41.59)	23737 (46.24)	24852 (64.53)	42752 (68.14%)	52579 (45.73)	205696 (45.3)
Physical activity							
Physically inactive (MET < 600)	46499 (27.35)	8313 (32.68)	19569 (37.59)	6263 (16.08)	13552 (21.5)	38512 (33.04)	333355 (28.5)
Physically active (MET >= 600)	123492 (72.65)	17121 (67.32)	32492 (62.41)	32692 (83.92)	49492 (78.5)	78066 (66.96)	132706 (71.5)

© 2025 Ahmed KY et al. *JAMA Network Open*.

eTable 3. Risk Factors Associated With Abdominal Obesity by Geographic Region

Characteristic	Africa (N = 175,156)<br>OR (95% CI)	Americas [N = 25,434]<br>OR (95% CI)	Eastern Mediterranean [N = 52,061]<br>OR (95% CI)	Europe [N = 38,955]<br>OR (95% CI)	South-East Asian Region [N = 63,044]<br>OR (95% CI)	Western Pacific [N = 116,578]<br>OR (95% CI)	Global [N = 471,228]<br>OR (95% CI)
Age Group							
15-29 years	1.00	1.00	1.00	1.00	1.00	—	1.00
30-44 years	1.94 (1.78, 2.11)	2.90 (2.67, 3.15)	2.58 (2.49, 2.66)	3.26 (3.01, 3.53)	2.61 (.50, 2.73)	3.53 (3.34, 3.73)	2.63 (2.58, 2.69)
45-59 years	2.65 (2.43, 2.90)	4.14 (3.80, 4.51)	4.09 (3.94, 4.25)	7.86 (7.26, 8.51)	3.46 (3.30, 3.62)	6.06 (5.69, 6.47)	4.17 (4.08, 4.27)
60+ years	3.06 (2.72, 3.44)	4.53 (4.08, 5.03)	4.19 (3.98, 4.41)	10.1 (9.22, 11.1)	3.20 (2.99, 3.43)	6.17 (5.66, 6.73)	4.53 (4.40, 4.67)
Sex							
Males	1.00	1.00	1.00	1.00	1.00	—	1.00
Females	4.55 (4.25, 4.86)	6.71 (6.33, 7.12)	9.66 (9.35, 9.98)	2.82 (2.65, 3.00)	4.52 (4.36, 4.68)	3.98 (3.75, 4.21)	5.44 (5.35, 5.54)
Educational Status							
No formal schooling	1.00	1.00	1.00	1.00	1.00	—	1.00
Primary or less	0.91 (0.79, 1.04)	2.20 (2.02, 2.39)	1.41 (1.36, 1.45)	0.78 (0.49, 1.21)	1.49 (1.41, 1.57)	1.60 (1.50, 1.69)	1.53 (1.50, 1.57)
Secondary or more	0.64 (0.56, 0.73)	3.64 (3.33, 3.98)	2.06 (1.99, 2.13)	0.79 (0.50, 1.22)	2.47 (2.35, 2.60)	1.72 (1.62, 1.84)	2.38 (2.33, 2.43)
Occupation							
Working	1.00	1.00	1.00	1.00	1.00	—	1.00
Not working	1.20 (1.11, 1.30)	0.88 (0.83, 0.93)	1.02 (0.99, 1.05)	1.05 (0.99, 1.11)	1.36 (1.31, 1.41)	1.42 (1.34, 1.50)	1.25 (1.23, 1.27)
Current Smoking							
Yes	1.00	1.00	1.00	1.00	1.00	—	1.00
No	1.30 (1.19, 1.42)	1.68 (1.57, 1.81)	1.50 (1.43, 1.58)	1.46 (1.36, 1.56)	1.30 (1.25, 1.35)	1.14 (1.07, 1.21)	1.10 (1.08, 1.13)
Fruit and Vegetable Intake							
Yes	1.00	1.00	1.00	1.00	1.00	—	1.00
No	0.88 (0.83, 0.94)	1.06 (1.01, 1.12)	1.26 (1.23, 1.30)	0.94 (0.89, 0.99)	1.03 (1.00, 1.07)	1.04 (0.99, 1.08)	1.22 (1.20, 1.24)
Physical Activity							
Yes	1.00	1.00	1.00	1.00	1.00	—	1.00
No	1.15 (1.07, 1.23)	1.53 (1.44, 1.63)	1.55 (1.50, 1.60)	1.10 (1.02, 1.18)	1.74 (1.68, 1.80)	1.33 (1.27, 1.39)	1.60 (1.57, 1.63)

OR = Odds Ratio; CI = Confidence Interval; SEAR = South-East Asia Region

Footnote: All associations between abdominal obesity and risk factors were adjusted for all other covariates, including age, gender, educational status, employment, smoking, alcohol use, fruit and vegetable intake, and physical activity

© 2025 Ahmed KY et al. *JAMA Network Open*.

eTable 4. Associations Between Different Patterns of Obesity and Diabetes and Hypertension Across 6 WHO Regions Globally From 2000 to 2020

**WHO Regions**	**WC and BMI relationships**	**Diabetes**		**Hypertension**	
		**OR (95% CI)**	**Evalue points**	**OR (95% CI)**	**Evalue points**
Global	Normal BMI	Ref	Ref	Ref	Ref
	Overweight	1.51 (1.46, 1.57)	1.23	1.31 (1.29, 1.34)	1.14
	Obesity	2.57 (2.49, 2.66)	1.60	2.88 (2.82, 2.93)	1.70
	Low WC	Ref	Ref	Ref	Ref
	High WC	2.30 (2.23, 2.37)	1.52	1.58 (1.55, 1.61)	1.26
	Low WC and Normal BMI	Ref	Ref	Ref	Ref
	High WC and Normal BMI	1.81 (1.72, 1.90)	1.35	1.29 (1.25, 1.33)	1.14
	Low WC and Overweight	1.36 (1.28, 1.45)	1.17	1.21 (1.18, 1.25)	1.10
	High WC and Overweight	2.17 (2.08, 2.26)	1.47	1.47 (1.43, 1.50)	1.21
	Low WC and Obesity	1.83 (1.63, 2.06)	1.35	1.39 (1.31, 1.49)	1.18
	High WC and Obesity	3.13 (3.01, 3.26)	1.77	2.06 (2.01, 2.10)	1.44
Africa	Normal BMI	Ref	Ref	Ref	Ref
	Overweight	1.36 (1.28, 1.44)	1.17	1.27 (1.23, 1.31)	1.13
	Obesity	1.64 (1.54, 1.76)	1.28	2.03 (1.95, 2.11)	1.42
	Low WC	Ref	Ref	Ref	Ref
	High WC	1.78 (1.69, 1.88)	1.33	1.45 (1.40, 1.50)	1.20
	Low WC and Normal BMI	Ref	Ref	Ref	Ref
	High WC and Normal BMI	1.55 (1.44, 1.67)	1.24	1.29 (1.24, 1.35)	1.14
	Low WC and Overweight	1.18 (1.07, 1.31)	1.09	1.23 (1.16, 1.30)	1.11
	High WC and Overweight	1.83 (1.71, 1.96)	1.35	1.44 (1.38, 1.51)	1.20
	Low WC and Obesity	1.41 (1.15, 1.72)	1.19	1.08 (0.97, 1.21)	1.04
	High WC and Obesity	2.18 (2.02, 2.34)	1.48	1.81 (1.73, 1.90)	1.35
Americas	Normal BMI	Ref	Ref	Ref	Ref
	Overweight	1.28 (1.05, 1.56)	1.13	1.51 (1.37, 1.66)	1.23
	Obesity	7.22 (6.1, 8.58)	2.69	6.21 (5.68, 6.79)	2.49
	Low WC	Ref	Ref	Ref	Ref

© 2025 Ahmed KY et al. *JAMA Network Open*.

	High WC	1.47 (1.24, 1.76)	1.21	1.42 (1.30, 1.54)	1.19
	Low WC and Normal BMI	Ref	Ref	Ref	Ref
	High WC and Normal BMI	1.62 (1.17, 2.22)	1.27	1.63 (1.38, 1.92)	1.28
	Low WC and Overweight	1.44 (1.06, 1.95)	1.20	1.75 (1.52, 2.01)	1.32
	High WC and Overweight	1.42 (1.11, 1.81)	1.19	1.62 (1.43, 1.83)	1.27
	Low WC and Obesity	2.93 (1.86, 4.51)	1.71	3.66 (3.07, 4.35)	1.91
	High WC and Obesity	2.35 (1.87, 2.96)	1.53	2.68 (2.39, 3.00)	1.64
Eastern Mediterranean	Normal BMI	Ref	Ref	Ref	Ref
	Overweight	1.49 (1.35, 1.64)	1.22	1.45 (1.37, 1.54)	1.20
	Obesity	2.31 (2.11, 2.54)	1.52	2.47 (2.33, 2.61)	1.57
	Low WC	Ref		Ref	Ref
	High WC	2.10 (1.91, 2.31)	1.45	1.67 (1.58, 1.76)	1.29
	Low WC and Normal BMI	Ref	Ref	Ref	Ref
	High WC and Normal BMI	1.76 (1.51, 2.05)	1.33	1.36 (1.24, 1.48)	1.17
	Low WC and Overweight	1.48 (1.24, 1.77)	1.22	1.36 (1.24, 1.50)	1.17
	High WC and Overweight	2.11 (1.86, 2.39)	1.45	1.69 (1.58, 1.82)	1.30
	Low WC and Obesity	2.32 (1.72, 3.08)	1.52	1.75 (1.47, 2.08)	1.32
	High WC and Obesity	3.15 (2.80, 3.56)	1.77	2.30 (2.14, 2.46)	1.52
Europe	Normal BMI	Ref	Ref	Ref	Ref
	Overweight	1.40 (1.23, 1.60)	1.18	1.60 (1.50, 1.70)	1.26
	Obesity	2.39 (2.12, 2.71)	1.55	3.02 (2.83, 3.21)	1.74
	Low WC	Ref	Ref	Ref	Ref
	High WC	2.15 (1.91, 2.43)	1.47	1.89 (1.78, 2.01)	1.37
	Low WC and Normal BMI	Ref	Ref	Ref	Ref
	High WC and Normal BMI	1.56 (1.26, 1.93)	1.25	1.25 (1.12, 1.39)	1.12
	Low WC and Overweight	1.26 (1.01, 1.57)	1.12	1.35 (1.22, 1.49)	1.16
	High WC and Overweight	1.86 (1.58, 2.20)	1.36	1.86 (1.72, 2.01)	1.36
	Low WC and Obesity	1.57 (0.99, 2.39)	1.25	2.23 (1.80, 2.77)	1.49
	High WC and Obesity	3.09 (2.66, 3.62)	1.76	2.85 (2.64, 3.07)	1.69
Southeast Asia	Normal BMI	Ref	Ref	Ref	Ref

© 2025 Ahmed KY et al. *JAMA Network Open*.

	Overweight	1.72 (1.57, 1.88)	1.31	2.91 (2.63, 3.23)	1.71
	Obesity	2.02 (1.77, 2.29)	1.42	8.92 (7.98, 9.99)	2.99
	Low WC	Ref	Ref	Ref	Ref
	High WC	2.71 (2.47, 2.97)	1.65	2.10 (1.97, 2.24)	1.45
	Low WC and Normal BMI	Ref	Ref	Ref	Ref
	High WC and Normal BMI	2.49 (2.20, 2.82)	1.58	1.78 (1.64, 1.94)	1.33
	Low WC and Overweight	1.71 (1.45, 2.01)	1.31	1.66 (1.50, 1.84)	1.29
	High WC and Overweight	3.07 (2.74, 3.44)	1.75	2.25 (2.08, 2.43)	1.50
	Low WC and Obesity	1.68 (1.08, 2.49)	1.30	2.01 (1.56, 2.58)	1.42
	High WC and Obesity	3.39 (2.94, 3.9)	1.84	3.20 (2.97, 3.64)	1.79
Western Pacific	Normal BMI	Ref	Ref	Ref	Ref
	Overweight	2.33 (2.15, 2.53)	1.53	0.81 (0.74, 0.88)	0.90
	Obesity	4.01 (3.72, 4.33)	2.00	2.24 (2.06, 2.44)	1.50
	Low WC	Ref	Ref	Ref	Ref
	High WC	3.63 (3.39, 3.90)	1.91	1.34 (1.29, 1.40)	1.16
	Low WC and Normal BMI	Ref	Ref	Ref	Ref
	High WC and Normal BMI	2.45 (2.11, 2.85)	1.57	0.86 (0.79, 0.94)	0.93
	Low WC and Overweight	2.06 (1.82, 2.32)	1.44	0.90 (0.85, 0.96)	0.95
	High WC and Overweight	3.63 (3.30, 4.00)	1.91	1.03 (0.98, 1.09)	1.01
	Low WC and Obesity	3.00 (2.36, 3.78)	1.73	0.99 (0.86, 1.15)	0.99
	High WC and Obesity	5.42 (4.99, 5.90)	2.33	1.57 (1.50, 1.64)	1.25

Footnote: All associations between obesity patterns and cardiometabolic outcomes were adjusted for covariates, including age, gender, educational status, employment, smoking, alcohol use, fruit and vegetable intake, and physical activity

© 2025 Ahmed KY et al. *JAMA Network Open*.

eTable 5. Associations Between Different Patterns of Obesity and Total Cholesterol and Triglyceride Across 6 WHO Regions Globally From 2000 to 2020

WHO Regions	WC and BMI relationships	High total cholesterol		High triglycerides	
		OR (95% CI)	Evalue point	OR (95% CI)	Evalue point
Global	Normal BMI	Ref	Ref	Ref	Ref
	Overweight	1.39 (1.35, 1.42)	1.18	1.29 (1.24, 1.33)	1.14
	Obesity	1.49 (1.45, 1.53)	1.22	1.54 (1.48, 1.59)	1.24
	Low WC	Ref	Ref	Ref	Ref
	High WC	1.49 (1.46, 1.53)	1.22	1.60 (1.55, 1.66)	1.26
	Low WC and Normal BMI	Ref	Ref	Ref	Ref
	High WC and Normal BMI	1.39 (1.35, 1.44)	1.18	1.56 (1.48, 1.64)	1.25
	Low WC and Overweight	1.40 (1.35, 1.46)	1.18	1.28 (1.21, 1.36)	1.13
	High WC and Overweight	1.65 (1.6, 1.69)	1.28	1.60 (1.53, 1.67)	1.26
	Low WC and Obesity	1.43 (1.31, 1.56)	1.20	1.43 (1.26, 1.63)	1.20
	High WC and Obesity	1.64 (1.6, 1.69)	1.28	1.85 (1.78, 1.93)	1.36
Africa	Normal BMI	Ref	Ref	Ref	Ref
	Overweight	1.34 (1.29, 1.40)	1.16	1.08 (1.00, 1.16)	1.04
	Obesity	1.48 (1.41, 1.56)	1.22	1.04 (0.95, 1.13)	1.02
	Low WC	Ref	Ref	Ref	ref
	High WC	1.58 (1.52, 1.64)	1.26	1.32 (1.24, 1.41)	1.15
	Low WC and Normal BMI	Ref	Ref	Ref	Ref
	High WC and Normal BMI	1.41 (1.34, 1.48)	1.19	1.43 (1.31, 1.55)	1.20
	Low WC and Overweight	1.23 (1.14, 1.32)	1.11	1.03 (0.90, 1.17)	1.01
	High WC and Overweight	1.66 (1.58, 1.74)	1.29	1.32 (1.21, 1.44)	1.15
	Low WC and Obesity	1.02 (0.86, 1.20)	1.01	1.17 (0.92, 1.49)	1.08
	High WC and Obesity	1.82 (1.73, 1.93)	1.35	1.21 (1.09, 1.33)	1.10
Americas	Normal BMI	Ref	Ref	Ref	Ref
	Overweight	1.38 (1.23, 1.56)	1.17	1.70 (1.36, 2.13)	1.30
	Obesity	3.09 (2.76, 3.47)	1.76	2.35 (1.88, 2.95)	1.53
	Low WC	Ref	Ref	Ref	Ref
	High WC	1.15 (1.04, 1.28)	1.07	2.91 (2.35, 3.60)	1.71

© 2025 Ahmed KY et al. *JAMA Network Open*.

	Low WC and Normal BMI	Ref	Ref	Ref	
	High WC and Normal BMI	1.2 (0.99, 1.45)	1.10	2.59 (1.81, 3.70)	1.61
	Low WC and Overweight	1.65 (1.38, 1.97)	1.28	1.34 (0.92, 1.93)	1.16
	High WC and Overweight	1.41 (1.20, 1.62)	1.19	2.92 (2.23, 3.84)	1.71
	Low WC and Obesity	6.19 (4.37, 8.89)	2.49	1.38 (0.65, 2.70)	1.17
	High WC and Obesity	1.68 (1.46, 1.93)	1.30	3.6 (2.78, 4.69)	1.90
Eastern Mediterranean	Normal BMI	Ref	Ref	Ref	Ref
	Overweight	1.58 (1.48, 1.69)	1.26	1.77 (1.63, 1.93)	1.33
	Obesity	1.84 (1.72, 1.97)	1.36	2.61 (2.40, 2.84)	1.62
	Low WC	Ref	Ref	Ref	Ref
	High WC	1.65 (1.55, 1.76)	1.28	2.09 (1.93, 2.26)	1.45
	Low WC and Normal BMI	Ref	Ref	Ref	Ref
	High WC and Normal BMI	1.61 (1.45, 1.78)	1.27	1.60 (1.39, 1.84)	1.26
	Low WC and Overweight	1.98 (1.77, 2.22)	1.41	1.81 (1.57, 2.08)	1.35
	High WC and Overweight	2.03 (1.87, 2.21)	1.42	2.40 (2.15, 2.67)	1.55
	Low WC and Obesity	2.46 (2.00, 3.03)	1.57	2.65 (2.06, 3.39)	1.63
	High WC and Obesity	2.37 (2.18, 2.57)	1.54	3.41 (3.07, 3.78)	1.85
Europe	Normal BMI	Ref	Ref	Ref	Ref
	Overweight	1.40 (1.29, 1.52)	1.18	1.16 (1.01, 1.33)	1.08
	Obesity	1.52 (1.39, 1.65)	1.23	1.43 (1.24, 1.64)	1.20
	Low WC	Ref	Ref	Ref	Ref
	High WC	1.44 (1.33, 1.55)	1.20	1.28 (1.13, 1.46)	1.13
	Low WC and Normal BMI	Ref	Ref	Ref	Ref
	High WC and Normal BMI	1.22 (1.06, 1.39)	1.10	1.23 (0.99, 1.54)	1.11
	Low WC and Overweight	1.33 (1.17, 1.52)	1.15	1.19 (0.95, 1.49)	1.09
	High WC and Overweight	1.57 (1.42, 1.74)	1.25	1.25 (1.05, 1.48)	1.12
	Low WC and Obesity	1.09 (0.78, 1.50)	1.04	1.51 (0.99, 2.30)	1.23
	High WC and Obesity	1.7 (1.54, 1.87)	1.30	1.55 (1.32, 1.82)	1.24
Southeast Asia	Normal BMI	Ref	Ref	Ref	Ref
	Overweight	1.51 (1.42, 1.61)	1.23	2.12 (1.94, 2.32)	1.46

© 2025 Ahmed KY et al. *JAMA Network Open*.

	Obesity	1.74 (1.58, 1.92)	1.32	2.16 (1.88, 2.49)	1.47
	Low WC	Ref	Ref	Ref	Ref
	High WC	1.64 (1.55, 1.74)	1.28	3.09 (2.84, 3.36)	1.76
	Low WC and Normal BMI	Ref	Ref	Ref	Ref
	High WC and Normal BMI	1.41 (1.30, 1.52)	1.19	2.95 (2.63, 3.32)	1.72
	Low WC and Overweight	1.63 (1.47, 1.80)	1.28	2.16 (1.90, 2.47)	1.47
	High WC and Overweight	1.87 (1.73, 2.02)	1.37	3.74 (3.34, 4.18)	1.93
	Low WC and Obesity	1.36 (1.03, 1.78)	1.17	1.59 (1.12, 2.22)	1.26
	High WC and Obesity	2.19 (1.97, 2.43)	1.48	3.51 (3.01, 4.10)	1.87
Western Pacific	Normal BMI	Ref	Ref	Ref	Ref
	Overweight	1.37 (1.30, 1.44)	1.17	1.53 (1.40, 1.67)	1.24
	Obesity	1.23 (1.17, 1.29)	1.11	2.07 (1.90, 2.26)	1.44
	Low WC	Ref	Ref	Ref	Ref
	High WC	1.44 (1.37, 1.50)	1.20	1.84 (1.70, 2.00)	1.36
	Low WC and Normal BMI	Ref	Ref	Ref	Ref
	High WC and Normal BMI	1.67 (1.52, 1.83)	1.29	1.40 (1.20, 1.63)	1.18
	Low WC and Overweight	1.37 (1.27, 1.47)	1.17	1.39 (1.22, 1.58)	1.18
	High WC and Overweight	1.7 (1.59, 1.80)	1.30	1.83 (1.65, 2.02)	1.35
	Low WC and Obesity	1.17 (0.97, 1.41)	1.08	1.57 (1.10, 2.20)	1.25
	High WC and Obesity	1.44 (1.37, 1.52)	1.20	2.36 (2.14, 2.60)	1.54

Footnote: All associations between obesity patterns and cardiometabolic outcomes were adjusted for covariates, including age, gender, educational status, employment, smoking, alcohol use, fruit and vegetable intake, and physical activity

© 2025 Ahmed KY et al. *JAMA Network Open*.

eTable 6. Interaction of Sex, Age, and Educational Status With the Associations Between Abdominal Obesity and Cardiometabolic Conditions, 2000 to 2020

WHO Regions	Variables	Categories	Diabetes	Hypertension	Total cholesterol	Triglycerides
			OR (95% CI)*	OR (95% CI)*	OR (95% CI)*	OR (95% CI)*
Global	Sex	Male	2.0 (1.91, 2.08)	1.55 (1.51, 1.60)	1.50 (1.45, 1.55)	1.85 (1.76, 1.95)
		Female	2.2 (2.10, 2.31)	1.66 (1.62, 1.71)	1.51 (1.47, 1.56)	1.44 (1.38, 1.51)
	Age group	18-29 years	1.31 (1.20, 1.43)	1.46 (1.40, 1.52)	1.48 (1.40, 1.56)	1.63 (1.51, 1.76)
		30-44 years	1.78 (1.69, 1.88)	1.50 (1.46, 1.55)	1.37 (1.32, 1.42)	1.60 (1.52, 1.69)
		45-59 years	2.75 (2.62, 2.89)	1.64 (1.60, 1.69)	1.56 (1.50, 1.61)	1.76 (1.66, 1.86)
		60+ years	3.03 (2.82, 3.25)	1.84 (1.76, 1.93)	1.63 (1.55, 1.72)	1.55 (1.42, 1.69)
	Educational status	No formal schooling	1.61 (1.51, 1.71)	1.62 (1.56, 1.69)	1.42 (1.36, 1.49)	1.67 (1.55, 1.79)
		Primary or less	2.56 (2.44, 2.70)	1.66 (1.61, 1.71)	1.70 (1.64, 1.75)	1.71 (1.62, 1.80)
		Secondary or more	2.23 (2.12, 2.35)	1.54 (1.50, 1.58)	1.42 (1.37, 1.47)	1.54 (1.46, 1.62)
Africa	Sex	Male	1.87 (1.71, 2.04)	1.48 (1.40, 1.57)	1.59 (1.48, 1.71)	1.83 (1.61, 2.09)
		Female	1.82 (1.69, 1.95)	1.38 (1.32, 1.44)	1.61 (1.53, 1.68)	1.18 (1.09, 1.28)
	Age group	18-29 years	1.29 (1.13, 1.46)	1.11 (1.03, 1.20)	1.43 (1.31, 1.55)	1.89 (1.62, 2.19)
		30-44 years	1.42 (1.31, 1.55)	1.42 (1.35, 1.50)	1.48 (1.40, 1.57)	1.55 (1.40, 1.72)
		45-59 years	2.22 (2.04, 2.42)	1.63 (1.55, 1.72)	1.74 (1.63, 1.85)	1.37 (1.23, 1.53)
		60+ years	2.82 (2.49, 3.20)	1.63 (1.50, 1.76)	1.77 (1.61, 1.96)	1.17 (0.99, 1.39)
	Educational status	No formal schooling	1.38 (1.27, 1.50)	1.60 (1.51, 1.69)	1.46 (1.36, 1.56)	1.88 (1.66, 2.12)
		Primary or less	2.31 (2.13, 2.52)	1.53 (1.45, 1.61)	1.69 (1.59, 1.79)	1.36 (1.24, 1.50)
		Secondary or more	1.95 (1.77, 2.16)	1.20 (1.13, 1.27)	1.67 (1.55, 1.79)	1.25 (1.10, 1.42)
Americas	Sex	Male	1.35 (0.96, 1.88)	1.77 (1.52, 2.06)	1.76 (1.42, 2.17)	3.79 (1.99, 7.23)
		Female	1.32 (0.92, 1.89)	1.15 (0.98, 1.35)	1.42 (1.16, 1.75)	3.00 (1.54, 5.83)
	Age group	18-29 years	1.21 (0.60, 2.43)	1.50 (1.17, 1.94)	2.78 (2.07, 3.72)	3.26 (1.54, 6.87)
		30-44 years	1.28 (0.87, 1.88)	1.27 (1.06, 1.51)	1.26 (1.00, 1.58)	2.76 (1.40, 5.45)
		45-59 years	1.51 (1.08, 2.11)	1.26 (1.07, 1.47)	1.49 (1.19, 1.87)	3.90 (1.98, 7.68)
		60+ years	1.35 (0.93, 1.96)	1.74 (1.41, 2.14)	1.20 (0.90, 1.59)	3.67 (1.68, 8.03)
	Educational status	No formal schooling	1.17 (0.57, 2.42)	1.37 (0.98, 1.92)	2.82 (1.74, 4.56)	4.58 (0.76, 27.62)
		Primary or less	1.55 (1.17, 2.04)	1.52 (1.34, 1.73)	1.52 (1.29, 1.78)	2.97 (21.8, 4.06)

© 2025 Ahmed KY et al. *JAMA Network Open*.

		Secondary or more	1.30 (0.98, 1.72)	1.39 (1.24, 1.57)	0.93 (0.80, 1.08)	2.81 (2.08, 3.79)
Eastern Mediterranean	Sex	Male	1.90 (1.68, 2.16)	1.70 (1.58, 1.83)	1.80 (1.64, 1.98)	2.29 (2.04, 2.56)
		Female	2.53 (2.13, 3.00)	1.67 (1.54, 1.82)	1.66 (1.51, 1.84)	2.19 (1.91, 2.52)
	Age group	18-29 years	1.62 (1.27, 2.07)	1.53 (1.37, 1.70)	1.97 (1.72, 2.25)	2.33 (1.96, 2.78)
		30-44 years	2.30 (1.91, 2.77)	1.58 (1.44, 1.72)	1.54 (1.39, 1.71)	2.02 (1.76, 2.32)
		45-59 years	2.33 (1.99, 2.73)	1.82 (1.65, 2.01)	1.60 (1.42, 1.80)	2.40 (2.05, 2.80)
		60+ years	2.66 (2.14, 3.29)	1.85 (1.61, 2.13)	1.86 (1.54, 2.23)	2.23 (1.77, 2.82)
	Educational status	No formal schooling	2.54 (2.08, 3.12)	1.59 (1.44, 1.75)	1.82 (1.57, 2.11)	2.75 (2.21, 3.41)
		Primary or less	1.92 (1.65, 2.23)	1.79 (1.64, 1.96)	1.91 (1.72, 2.12)	2.14 (1.89, 2.42)
		Secondary or more	21.6 (1.83, 2.54)	1.69 (1.54, 1.85)	1.49 (1.35, 1.65)	1.92 (1.68, 2.18)
Europe	Sex	Male	1.88 (0.88, 4.01)	1.28 (0.95, 1.72)	1.65 (1.03, 2.64)	1.76 (0.79, 3.89)
		Female	2.26 (1.06, 4.84)	1.78 (1.33, 2.39)	1.78 (1.12, 2.84)	1.40 (0.64, 3.05)
	Age group	18-29 years	1.44 (0.59, 3.53)	1.66 (1.20, 2.30)	1.77 (1.05, 3.00)	1.54 (0.68, 3.52)
		30-44 years	1.85 (0.85, 4.01)	1.28 (0.95, 1.74)	1.62 (1.01, 2.61)	1.57 (0.70, 3.50)
		45-59 years	2.71 (1.27, 5.81)	1.48 (1.10, 2.00)	1.82 (1.14, 2.92)	1.70 (0.77, 3.77)
		60+ years	2.51 (1.17, 5.35)	1.64 (1.20, 2.23)	1.64 (1.02, 2.64)	1.48 (0.65, 3.33)
	Educational status	No formal schooling	1.93 (0.22, 17,3)	0.90 (0.39, 2.09)	2.11 (0.54, 8.22)	1.56 (0.24, 10.33)
		Primary or less	2.43 (1.60, 3.68)	1.99 (1.68, 2.37)	1.71 (1.38, 2.12)	1.89 (0.50, 7.20)
		Secondary or more	1.87 (1.58, 2.20)	1.92 (1.79, 2.05)	1.40 (1.27, 1.54)	1.31 (1.14, 1.51)
Southeast Asia	Sex	Male	2.29 (1.91, 2.74)	1.98 (1.76, 2.22)	1.76 (1.56, 1.99)	2.79 (2.34, 3.33)
		Female	2.76 (2.34, 3.25)	2.21 (2.02, 2.42)	1.82 (1.68, 1.98)	3.12 (2.77, 3.51)
	Age group	18-29 years	2.36 (1.56, 3.58)	2.41 (1.99, 2.93)	2.29 (1.91, 2.74)	3.42 (2.64, 4.42)
		30-44 years	2.32 (1.95, 2.75)	2.08 (1.87, 2.31)	1.69 (1.52, 1.87)	2.96 (2.58, 3.41)
		45-59 years	2.64 (2.28, 3.05)	1.90 (1.72, 2.10)	1.58 (1.43, 1.74)	2.82 (2.46, 3.24)
		60+ years	2.76 (2.28, 3.33)	2.01 (1.75, 2.31)	1.69 (1.47, 1.96)	2.66 (2.08, 3.39)
	Educational status	No formal schooling	2.39 (1.82, 3.15)	2.04 (1.73, 2.40)	2.14 (1.82, 2.52)	3.13 (2.58, 3.79)
		Primary or less	3.02 (2.59, 3.52)	2.28 (2.08, 2.51)	1.84 (1.68, 2.01)	3.38 (2.95, 3.87)
		Secondary or more	2.19 (1.86, 2.59)	1.97 (1.78, 2.18)	1.46 (1.32, 1.62)	2.44 (2.06, 2.87)
Western Pacific	Sex	Male	3.25 (2.93, 3.62)	1.20 (1.12, 1.28)	1.69 (1.57, 1.83)	2.16 (1.89, 2.47)
		Female	3.68 (3.21, 4.21)	1.79 (1.66, 1.93)	1.51 (1.40, 1.63)	1.53 (1.32, 1.77)

© 2025 Ahmed KY et al. *JAMA Network Open*.

	Age group	18-29 years	1.93 (1.59, 2.35)	1.75 (1.60, 1.93)	1.64 (1.45, 1.85)	1.69 (1.40, 2.04)
		30-44 years	3.38 (2.97, 3.86)	1.33 (1.23, 1.43)	1.42 (1.31, 1.54)	1.62 (1.40, 1.87)
		45-59 years	4.62 (4.13, 5.18)	1.35 (1.25, 1.44)	1.61 (1.49, 1.74)	1.88 (1.61, 2.19)
		60+ years	4.73 (3.95, 5.67)	1.47 (1.31, 1.65)	1.75 (1.54, 2.00)	2.14 (1.67, 2.76)
	Educational status	No formal schooling	3.47 (2.85, 4.22)	1.42 (1.24, 1.63)	1.78 (1.54, 2.07)	1.93 (1.49, 2.50)
		Primary or less	3.94 (3.48, 4.45)	1.56 (1.45, 1.66)	1.84 (1.71, 1.99)	1.62 (1.37, 1.92)
		Secondary or more	3.03 (2.73, 3.37)	1.42 (1.35, 1.50)	1.25 (1.17, 1.33)	1.93 (1.75, 2.14)

Footnote: All models between obesity patterns and cardiometabolic outcomes were adjusted for covariates, including age, gender, educational status, employment, smoking, alcohol use, fruit and vegetable intake, and physical activity; *Adjusted odds ratios by subgroup were estimated using the emmeans package in R through marginal effects analysis.

© 2025 Ahmed KY et al. *JAMA Network Open*.

eAppendix 1. Stata Codes for Data Cleaning

```
**********************************************************************
************Created by: Kedir Yimam Ahmed******************************
************Date: June 2024********************************************
************Project: Central obesity and cardiometabolid disorders Gllobal*****
**********************************************************************

clear
clear matrix

cd "C:\Users\kahmed07\Dropbox\RHRI documents\Central Obesity\Global\LMICs\Dataset"
use Afghanistan_2018.dta
drop if m8==1|m8==3

*********************************************************************
********survey weight for cheking the outcome variable****************
*********************************************************************
gen wgt = wstep1/10000
gen strata = stratum
svyset psu [pw = wgt], strata(stratum) vce(linearized)

*********************************************************************
************************central obesity******************************
*********************************************************************
ge cenobese = 0
replace cenobese = 1 if (m14>=94 & sex=="Men")|(m14>=80 & sex=="Women")
replace cenobese = . if m14 == .
lab def cenobese 0 "not central obesity" 1 "central obesity"
lab val cenobese cenobese
lab var cenobese "presence of central obesity"
ta cenobese
 *NB: The cut-off point is based on the WHO recommemndation of 94/80
 *(increased risk)*

*********************************************************************
************************Body mass index******************************
*********************************************************************
gen bmi = . if m11==888.8|m12==888.8
gen height_m = m11 / 100
replace bmi = m12 / (height_m^2)
label variable bmi "Body Mass Index (BMI)"

*********************************************************************
************************BMI categories*******************************
*********************************************************************
gen bmic=1 if bmi<18.5
replace bmic=2 if bmi>=18.5 & bmi<25
replace bmic=3 if bmi>=25 & bmi<30
replace bmic=4 if bmi>=30
label define bmic 1"Underweight" 2"Normal" 3"Overweight" 4 "Obese"
label values bmic bmic
tab bmic


*********************************************************************
*******************Composite WC and BMI index************************
*********************************************************************
gen cen_comp=1 if (bmic==1|bmic==2) & cenobese==0
```

© 2025 Ahmed KY et al. *JAMA Network Open*.

```
replace cen_comp=2 if (bmic==1|bmic==2) & cenobese==1
replace cen_comp=3 if (bmic==3) & cenobese==0
replace cen_comp=4 if (bmic==3) & cenobese==1
replace cen_comp=5 if (bmic==4) & cenobese==0
replace cen_comp=6 if (bmic==4) & cenobese==1
label define cen_comp 1"low WC and Low BMI" 2"High WC and low BMI" 3"Low WC and overweight" ///
4 "High WC and overweight" 5 "low WC and obese" 6 "High WC and obese"
label values cen_comp cen_comp
tab cen_comp

*******************************************************************
************************highest education completed*****************
*******************************************************************
tab c5, missing
ge edustatus = 1 if c5==1
replace edustatus = 2 if c5==2|c5==3
replace edustatus = 3 if c5==4|c5==5|c5==6
label define edustatus 1 "No formal schooling" 2 "Primary or less" ///
3 "Secondary or more"
label value edustatus edustatus
label variable edustatus "highest education completed"
tab edustatus

*******************************************************************
************************marital status******************************
*******************************************************************
tab c7, nol
gen maritalstat=1 if c7==1
replace maritalstat=2 if c7==2|c7==6
replace maritalstat=3 if c7==3|c7==4|c7==5
label define maritalstat 1"never married" 2"currently married/Cohabitating" ///
3"formerly married"
label value maritalstat maritalstat
label variable maritalstat "marital status"
tab maritalstat

*******************************************************************
************************age group***********************************
*******************************************************************
tab age, missing
summarize age
ge agegroup = 1 if age<30
replace agegroup = 2 if age>=30 & age<45
replace agegroup = 3 if age>=45 & age<60
replace agegroup = 4 if age>=60
label define agegroup 1 "18-29 years" 2 "30-44 years" 3 "45-59 years" 4 "60+ years"
label value agegroup agegroup
label variable agegroup "age group"
tab agegroup

*******************************************************************
************************occupational status**********************
*******************************************************************
tab c8, missing
ge occupation = 1 if c8==6|c8==7|c8==8|c8==9
replace occupation = 2 if c8==1|c8==2|c8==3|c8==4|c8==5
replace occupation = . if c8==88
label define occupation 1 "Not working" 2 "Working"
```

© 2025 Ahmed KY et al. *JAMA Network Open*.

```
label value occupation occupation
label variable occupation "Occupational status"
tab occupation

********************************************************************
************************smoking status************************
********************************************************************
ta t1, m
gen smoke_status=1 if t1==1
replace smoke_status=3 if t1==2
replace smoke_status=2 if t8==1
label define smoke_status 1 "current smoker" 2 "Ex-smoker" 3 "Never smoker"
label value smoke_status smoke_status
label variable smoke_status "smoking status"
tab smoke_status


********************************************************************
**************Number of drinks in the past 7 days********************
********************************************************************
tab1 a10a a10b a10c a10d a10e a10f a10g
replace a10a=. if a10a==77|a10a==88
replace a10b=. if a10b==77|a10b==88
replace a10c=. if a10c==77|a10c==88
replace a10d=. if a10d==77|a10d==88
replace a10e=. if a10e==77|a10e==88
replace a10f=. if a10f==77|a10f==88
replace a10g=. if a10g==77|a10g==88

gen alcohol_laswk=a10a+a10b+a10c+a10d+a10e+a10f+a10g
replace alcohol_laswk=0 if a1==2
ta alcohol_laswk
label variable alcohol_laswk "number of alcohol drinks last week"
tab alcohol_laswk

********************************************************************
************************fruit consumption***************************
********************************************************************
ge dailyfruit = 2 if d1 < 7
replace dailyfruit = 1 if d1==7
label define dailyfruit 1 "Yes" 2 "No"
label value dailyfruit dailyfruit
label variable dailyfruit "Daily fruit consumption"
tab dailyfruit

********************************************************************
************************vegetable consumption******************
********************************************************************
ge dailyveg = 2 if d3 < 7
replace dailyveg = 1 if d3==7
replace dailyveg = . if d3==77
label define dailyveg 1 "Yes" 2 "No"
label value dailyveg dailyveg
label variable dailyveg "Daily vegetable consumption"
tab dailyveg

********************************************************************
************************Fruit and vegetable consumption*************
```

```
*********************************************************************
ge fruit_veg = 2 if dailyveg==2|dailyfruit==2
replace fruit_veg = 1 if dailyveg==1|dailyfruit==1
label define fruit_veg 1 "Yes" 2 "No"
label value fruit_veg fruit_veg
label variable fruit_veg "Daily fruit or vegetable consumption"
tab fruit_veg


*********************************************************************
************************Fruit and vegetable serviungs per day********
*********************************************************************
tab1 d2 d4
replace d2=. if d2==99
replace d4=. if d4==99
ge fruit_veg_servings=d2+d4
ge fruitveg_5servings = 1 if fruit_veg_servings>=5
replace fruitveg_5servings = 2 if fruit_veg_servings<5
label define fruitveg_5servings 1 "Yes" 2 "No"
label value fruitveg_5servings fruitveg_5servings
label variable fruitveg_5servings "5 or more fruit or vegetable servings per day"
tab fruitveg_5servings


*********************************************************************
***********************Physical activity*****************************
*********************************************************************
gen p3 = (p3a*60)+p3b
gen p6 = (p6a*60)+p6b
gen p9 = (p9a*60)+p9b
gen p12 = (p12a*60)+p12b
gen p15 = (p15a*60)+p15b

ge MET1 = p2*p3*8
replace MET1 = . if p2==77

ge MET2 = p5*p6*4
replace MET2 = . if p5==77

ge MET3 = p8*p9*4
replace MET3 = . if p8==77

ge MET4 = p11*p12*8
replace MET4 = . if p11==77

ge MET5 = p14*p15*4
replace MET5 = . if p14==77

recode MET1 MET2 MET3 MET4 MET5 (missing = 0), prefix(new_)
ge MET_tot = new_MET1+new_MET2+new_MET3+new_MET4+new_MET5

ge physact = 2 if MET_tot < 600
replace physact = 1 if MET_tot >= 600
label define physact 1 "Yes" 2 "No"
label value physact physact
label variable physact "Physical activity"
ta physact
```

© 2025 Ahmed KY et al. *JAMA Network Open*.

```
**********************************************************************
************************Raised blood pressure************************
**********************************************************************
gen sysbp = (m4a+m5a+m6a)/3
gen diasbp = (m4b+m5b+m6b)/3
ge htn = . if m4a==888|m5a==888|m6a==888|m4b==888|m5b==888|m6b==888
replace htn = 1 if sysbp >= 140|diasbp >= 90|h3==1
replace htn = 0 if (sysbp < 140 & diasbp < 90)|h3==2
label define htn 1 "Yes" 0 "No"
label value htn htn
label variable htn "Presence of hypertension"
tab htn

**********************************************************************
************************Raised blood glucose*************************
**********************************************************************
gen diabetes = 0 if b5<126|h8==2
replace diabetes = 1 if b5 >= 126|h8==1
replace diabetes = . if b5==777|b5==.
label define diabetes 1 "Yes" 0 "No"
label value diabetes diabetes
label variable diabetes "Presence of diabetes"
tab diabetes

**********************************************************************
************************Raised cholestrol level**********************
**********************************************************************
gen cholestrol = 0 if b8<190|b9==2
replace cholestrol = 1 if b8 >= 190|b9==1
replace cholestrol = . if b8==777|b8==.|b9==77
label define cholestrol 1 "Yes" 0 "No"
label value cholestrol cholestrol
label variable cholestrol "Presence of high cholesterol"
tab cholestrol

*******************************************************************************************
**************denormilising and recoding country level clusters before pooling*************
*******************************************************************************************
gen WC_original=m14
gen height_original=m11
gen weight_original=m12
gen highestedu_original=c5
gen occupation_original=c8
gen martalstat_original=c7
gen current_smoking=t1
gen ever_alcohol=a1
gen proceeded_food=d7
gen salt_consumption=d8
gen past_smoker=t8
gen hrtattack_stroke=h17
gen aspirin=h18
gen statin=h19
gen sedentaryhrs_perday=p16a
gen sedentarymins_perday=p16b
gen creatinine=b15
gen FBS_mgdl = b5
gen cholestrol_mgdl = b8
```

© 2025 Ahmed KY et al. *JAMA Network Open*.

```
gen country="afghanistan_2018"
tostring agegroup, replace

keep agerange age agegroup sex WC_original height_original weight_original cen_comp ///
cenobese bmi height_m bmic highestedu_original edustatus occupation_original occupation martalstat_original ///
current_smoking smoke_status past_smoker ever_alcohol alcohol_laswk alcohol proceeded_food salt_consumption ///
fruit_veg_servings fruitveg_5servings dailyfruit dailyveg fruit_veg sedentaryhrs_perday sedentarymins_perday ///
creatinine MET_tot physact sysbp diasbp aspirin statin hrtattack_stroke ///
htn diabetes cholestrol FBS_mgdl cholestrol_mgdl country

save "C:\Users\kahmed07\Dropbox\RHRI documents\Central Obesity\Global\LMICs\Clean dataset\afghanistan_2018.dta",
replace
```

© 2025 Ahmed KY et al. *JAMA Network Open*.

eAppendix 2. R Codes for Regression Models

```
#################################################
##--------Author: Kedir Ahmed-----------########
##--------Project: Central obesity global---#####
#################################################
rm(list = ls())

##################################################
####loading the library and opening the datasets##
#loading relevant packages
library(haven)
library(gtsummary)
library(dplyr)
library(officer)
library(flextable)
library(broom)
library(flextable)
library(purrr)
library(emmeans)

#Opening the dataset
mydata <-
  read_dta("C:/Users/User/Dropbox/RHRI documents/Central Obesity/Global/LMICs/Analysis/Final_pooled_dateset.dta")
mydata <- mydata %>% mutate(across(where(is.labelled), as_factor))

#Checking the glimpse of the data
head (mydata)
names (mydata)

##############################################################
##--descriptive statistics to characterize the population----##
##############################################################

# List of variables to process
variables <- c("sex_recoded", "agegroup", "edustatus", "occupation",
```

© 2025 Ahmed KY et al. *JAMA Network Open*.

```
              "current_smoking_recoded", "alcohol_lastwk_recoded",
              "dailyfruit", "dailyveg", "recommended_fruitveg", "physact")

# Loop through each variable and generate the table with proportions
for (var in variables) {
  cat("\nVariable:", var, "\n")  # Print variable name as a header

  # Calculate frequency and proportion
  freq <- table(mydata[[var]])  # Frequency
  prop <- prop.table(freq)      # Proportion

  # Combine frequency and proportion into a data frame
  result <- data.frame(Frequency = as.integer(freq),
                       Proportion = round(100 * prop, 2)) # Convert to percentage
  print(result)
}

#################################################################
####------Re-coding triglycerides for analysis---------------##
#################################################################

# removing outliers
# Recoding triglyceride values to handle invalid values
mydata <- mydata %>%
  mutate(
    triyglycerides__mmol_recoded = ifelse(triyglycerides__mmol_recoded == 0 | triyglycerides__mmol_recoded > 100, NA,
triyglycerides__mmol_recoded),
    triyglycerides__mgdl_recoded = ifelse(triyglycerides__mgdl_recoded == 0 | triyglycerides__mgdl_recoded > 8000, NA,
triyglycerides__mgdl_recoded)
  )

# Define a function to recode triglycerides
recode_triglycerides <- function(mmol, mgdl) {
  if (is.na(mmol) && is.na(mgdl)) {
    return(NA)
  }
```

© 2025 Ahmed KY et al. *JAMA Network Open*.

```
  # Convert mg/dL to mmol/L if available, otherwise use mmol/L
  triglyceride_value <- if (!is.na(mgdl)) mgdl / 88.57 else mmol

  # Apply threshold
  if (triglyceride_value >= 1.7) {
    return("High")
  } else {
    return("Normal")
  }
}

# Create a Triglycerides_status column
mydata <- mydata %>%
  rowwise() %>%
  mutate(
    Triglycerides_status = recode_triglycerides(triyglycerides__mmol_recoded, triyglycerides__mgdl_recoded)
  ) %>%
  ungroup()

# Create a binary label for regression: 1 = High, 0 = Normal
mydata <- mydata %>%
  mutate(Triglycerides_binary = ifelse(Triglycerides_status == "High", 1,
                                       ifelse(Triglycerides_status == "Normal", 0, NA)))

# Verify the output
table(mydata$Triglycerides_status)
table(mydata$Triglycerides_binary)
names(mydata)

#################################################################
####------Regression Models for central obesity --------------##
################################################################
mydata$bmic_recoded <- relevel(mydata$bmic_recoded, ref = "Normal")

# Split the data by WHO_region and fit separate models for each region
```

© 2025 Ahmed KY et al. *JAMA Network Open*.

```
models_by_region <- mydata %>%
  group_split(WHO_region) %>%
  map(~ glm(
    cenobese_recoded ~ agegroup + sex_recoded + edustatus + occupation +
      current_smoking_recoded + recommended_fruitveg + physact,
    data = .x, family = binomial
  ))

# Get region names from the dataset
region_names <- unique(mydata$WHO_region)

# Create a gtsummary regression table for each region
tables_by_region <- map2(
  models_by_region,
  region_names,
  ~ tbl_regression(
    .x,
    exponentiate = TRUE, # Display Odds Ratios and 95% CIs
    label = list(
      agegroup ~ "Age Group",
      sex_recoded ~ "Sex",
      edustatus ~ "Educational Status",
      occupation ~ "Occupation",
      current_smoking_recoded ~ "Current Smoking",
      recommended_fruitveg ~ "Fruit & Vegetable Intake",
      physact ~ "Physical Activity"
    )
  ) %>%
    modify_header(label = paste0(.y, " Results")) %>% # Add region name as the header
    modify_table_body(
      fun = ~ .x %>%
        select(-p.value) # Remove the p-value column
    )
)
```

© 2025 Ahmed KY et al. *JAMA Network Open*.

```
# Combine all regional tables into one merged table
final_table <- tbl_merge(
  tbls = tables_by_region,         # List of tables to merge
  tab_spanner = region_names       # Column headers with region names
)

# Convert the merged table to a flextable for Word export
ft <- as_flex_table(final_table)

# Create a Word document and add the table
doc <- read_docx() %>%
  body_add_par("Logistic Regression Results by Region", style = "heading 1") %>%
  body_add_flextable(ft)

# Save the Word document
output_path <- "C:/Users/User/Dropbox/RHRI documents/Central Obesity/Global/LMICs/Analysis/Table_Regions.docx"
print(doc, target = output_path)

#################################################################
####------Regression Models for hypertension -----------------##
################################################################

# Define a function for regression analysis
run_model <- function(data, formula) {
  model <- glm(formula, data = data, family = binomial)  # Fit the model
  odds_ratios <- exp(coef(model))  # Calculate Odds Ratios
  conf_intervals <- exp(confint(model))  # 95% Confidence Intervals
  # Combine results into a data frame
  data.frame(
    Variable = names(odds_ratios),
    Odds_Ratio = odds_ratios,
    CI_Lower = conf_intervals[, 1],
    CI_Upper = conf_intervals[, 2]
  )
}
```

© 2025 Ahmed KY et al. *JAMA Network Open*.

```
# Global analysis
global_data <- mydata  # Use full dataset
results_global_WC <- run_model(global_data, htn ~ cenobese_recoded + agegroup +
                               sex_recoded + edustatus + occupation +
                               current_smoking_recoded + recommended_fruitveg + physact)
results_global_BMI <- run_model(global_data, htn ~ bmic_recoded + agegroup +
                                sex_recoded + edustatus + occupation +
                                current_smoking_recoded + recommended_fruitveg + physact)
results_global_composite <- run_model(global_data, htn ~ cen_comp_recoded + agegroup +
                                      sex_recoded + edustatus + occupation +
                                      current_smoking_recoded + recommended_fruitveg + physact)

# Regional analysis
run_regional_models <- function(region) {
  regional_data <- subset(mydata, WHO_region == region)
  list(
    WC = run_model(regional_data, htn ~ cenobese_recoded + agegroup + sex_recoded +
                   edustatus + occupation + current_smoking_recoded +
                   recommended_fruitveg + physact),
    BMI = run_model(regional_data, htn ~ bmic_recoded + agegroup + sex_recoded +
                    edustatus + occupation + current_smoking_recoded +
                    recommended_fruitveg + physact),
    Composite = run_model(regional_data, htn ~ cen_comp_recoded + agegroup + sex_recoded +
                          edustatus + occupation + current_smoking_recoded +
                          recommended_fruitveg + physact)
  )
}

# Run models for each region
results_africa <- run_regional_models("Africa")
results_americas <- run_regional_models("Americas")
results_EM <- run_regional_models("Eastern Mediterranean")
results_Europe <- run_regional_models("Europe")
results_SEARD <- run_regional_models("SEARD")
```

© 2025 Ahmed KY et al. *JAMA Network Open*.

```
results_WF <- run_regional_models("Western Pacific")


##################################################################
####------Regression Models for diabetes---------------------##
#################################################################
# Global analysis
results_global_WC_diabetes <- run_model(global_data, diabetes ~ cenobese_recoded + agegroup +
                          sex_recoded + edustatus + occupation +
                          current_smoking_recoded + recommended_fruitveg + physact)
results_global_BMI_diabetes <- run_model(global_data, diabetes ~ bmic_recoded + agegroup +
                          sex_recoded + edustatus + occupation +
                          current_smoking_recoded + recommended_fruitveg + physact)
results_global_composite_diabetes <- run_model(global_data, diabetes ~ cen_comp_recoded + agegroup +
                               sex_recoded + edustatus + occupation +
                               current_smoking_recoded + recommended_fruitveg + physact)

# Regional analysis
run_regional_models_diabetes <- function(region) {
  regional_data <- subset(mydata, WHO_region == region)
  list(
    WC = run_model(regional_data, diabetes ~ cenobese_recoded + agegroup + sex_recoded +
                 edustatus + occupation + current_smoking_recoded +
                 recommended_fruitveg + physact),
    BMI = run_model(regional_data, diabetes ~ bmic_recoded + agegroup + sex_recoded +
                  edustatus + occupation + current_smoking_recoded +
                  recommended_fruitveg + physact),
    Composite = run_model(regional_data, diabetes ~ cen_comp_recoded + agegroup + sex_recoded +
                        edustatus + occupation + current_smoking_recoded +
                        recommended_fruitveg + physact)
  )
}

# Run models for each region
results_africa_diabetes <- run_regional_models_diabetes("Africa")
```

© 2025 Ahmed KY et al. *JAMA Network Open*.

```
results_americas_diabetes <- run_regional_models_diabetes("Americas")
results_EM_diabetes <- run_regional_models_diabetes("Eastern Mediterranean")
results_Europe_diabetes <- run_regional_models_diabetes("Europe")
results_SEARD_diabetes <- run_regional_models_diabetes("SEARD")
results_WF_diabetes <- run_regional_models_diabetes("Western Pacific")

##################################################################
####------Regression Models for total cholesterol-------------##
#################################################################

# Global analysis
results_global_WC_totchol <- run_model(global_data, cholestrol ~ cenobese_recoded + agegroup +
                                       sex_recoded + edustatus + occupation +
                                       current_smoking_recoded + recommended_fruitveg + physact)
results_global_BMI_totchol <- run_model(global_data, cholestrol ~ bmic_recoded + agegroup +
                                        sex_recoded + edustatus + occupation +
                                        current_smoking_recoded + recommended_fruitveg + physact)
results_global_composite_totchol <- run_model(global_data, cholestrol ~ cen_comp_recoded + agegroup +
                                              sex_recoded + edustatus + occupation +
                                              current_smoking_recoded + recommended_fruitveg + physact)

# Regional analysis
run_regional_models_totchol <- function(region) {
  regional_data <- subset(mydata, WHO_region == region)
  list(
    WC = run_model(regional_data, cholestrol ~ cenobese_recoded + agegroup + sex_recoded +
                   edustatus + occupation + current_smoking_recoded +
                   recommended_fruitveg + physact),
    BMI = run_model(regional_data, cholestrol ~ bmic_recoded + agegroup + sex_recoded +
                    edustatus + occupation + current_smoking_recoded +
                    recommended_fruitveg + physact),
    Composite = run_model(regional_data, cholestrol ~ cen_comp_recoded + agegroup + sex_recoded +
                          edustatus + occupation + current_smoking_recoded +
                          recommended_fruitveg + physact)
  )
```

© 2025 Ahmed KY et al. *JAMA Network Open*.

```
}

# Run models for each region
results_africa_totchol <- run_regional_models_totchol("Africa")
results_americas_totchol <- run_regional_models_totchol("Americas")
results_EM_totchol <- run_regional_models_totchol("Eastern Mediterranean")
results_Europe_totchol <- run_regional_models_totchol("Europe")
results_SEARD_totchol <- run_regional_models_totchol("SEARD")
results_WF_totchol <- run_regional_models_totchol("Western Pacific")

##################################################################
####------Regression Models for total triglycerides-------------##
#################################################################

# Global analysis
results_global_WC_triglycerides <- run_model(global_data, Triglycerides_binary ~ cenobese_recoded + agegroup +
                                  sex_recoded + edustatus + occupation +
                                  current_smoking_recoded + recommended_fruitveg + physact)
results_global_BMI_triglycerides <- run_model(global_data, Triglycerides_binary ~ bmic_recoded + agegroup +
                                   sex_recoded + edustatus + occupation +
                                   current_smoking_recoded + recommended_fruitveg + physact)
results_global_composite_triglycerides <- run_model(global_data, Triglycerides_binary ~ cen_comp_recoded + agegroup +
                                        sex_recoded + edustatus + occupation +
                                        current_smoking_recoded + recommended_fruitveg + physact)

# Regional analysis
run_regional_models_triglycerides <- function(region) {
  regional_data <- subset(mydata, WHO_region == region)
  list(
    WC = run_model(regional_data, Triglycerides_binary ~ cenobese_recoded + agegroup + sex_recoded +
                 edustatus + occupation + current_smoking_recoded +
                 recommended_fruitveg + physact),
    BMI = run_model(regional_data, Triglycerides_binary ~ bmic_recoded + agegroup + sex_recoded +
                  edustatus + occupation + current_smoking_recoded +
                  recommended_fruitveg + physact),
```

© 2025 Ahmed KY et al. *JAMA Network Open*.

```
  Composite = run_model(regional_data, Triglycerides_binary ~ cen_comp_recoded + agegroup + sex_recoded +
                        edustatus + occupation + current_smoking_recoded +
                        recommended_fruitveg + physact)
 )
}

# Run models for each region
results_africa_triglycerides <- run_regional_models_triglycerides("Africa")
results_americas_triglycerides <- run_regional_models_triglycerides("Americas")
results_EM_triglycerides <- run_regional_models_triglycerides("Eastern Mediterranean")
results_Europe_triglycerides <- run_regional_models_triglycerides("Europe")
results_SEARD_triglycerides <- run_regional_models_triglycerides("SEARD")
results_WF_triglycerides <- run_regional_models_triglycerides("Western Pacific")

####################################################################
####------Writing a function for Interaction checks ----------##
###################################################################

run_emm_analysis <- function(data, outcome_var, region = NULL) {
 # Filter by region if provided
 if (!is.null(region)) {
   data <- data %>% filter(WHO_region == region)
 }

 # Dynamically build the model formula
 formula_str <- paste0(outcome_var, " ~ cenobese_recoded*agegroup + cenobese_recoded*sex_recoded + 
cenobese_recoded*edustatus + ",
                       "occupation + current_smoking_recoded + recommended_fruitveg + physact")
 model <- glm(as.formula(formula_str), data = data, family = binomial)

 # Helper function to evaluate emmeans contrasts
 get_emm_summary <- function(by_var) {
   # Build formula like: ~ cenobese_recoded | sex_recoded
   emm_formula <- as.formula(paste("~ cenobese_recoded |", by_var))
   emm <- emmeans(model, emm_formula)
```

© 2025 Ahmed KY et al. *JAMA Network Open*.

```
    contrast_res <- contrast(emm, method = "revpairwise", adjust = "none")
    summary(contrast_res, type = "response", infer = TRUE)
  }

  # Run emmeans for each grouping variable
  list(
    model = model,
    sex_summary = get_emm_summary("sex_recoded"),
    age_summary = get_emm_summary("agegroup"),
    edu_summary = get_emm_summary("edustatus")
  )
}

#################################################################
####-------------Interaction results for diabetes--------------##
#################################################################

# Diabetes global
results_diab_global <- run_emm_analysis(data = mydata, outcome_var = "diabetes")
results_diab_global$sex_summary
results_diab_global$age_summary
results_diab_global$edu_summary


# Diabetes Africa
results_diab_africa <- run_emm_analysis(data = mydata, outcome_var = "diabetes", region = "Africa")
results_diab_africa$sex_summary
results_diab_africa$age_summary
results_diab_africa$edu_summary

# Diabetes Americas
results_diab_americas <- run_emm_analysis(data = mydata, outcome_var = "diabetes", region = "Americas")
results_diab_americas$sex_summary
results_diab_americas$age_summary
results_diab_americas$edu_summary
```

© 2025 Ahmed KY et al. *JAMA Network Open*.

```
# Diabetes Eastern Mediterranean
results_diab_em <- run_emm_analysis(data = mydata, outcome_var = "diabetes", region = "Eastern Mediterranean")
results_diab_em $sex_summary
results_diab_em $age_summary
results_diab_em $edu_summary

# Diabetes Europe
results_diab_eu <- run_emm_analysis(data = mydata, outcome_var = "diabetes", region = "Europe")
results_diab_eu $sex_summary
results_diab_eu $age_summary
results_diab_eu $edu_summary

# Diabetes Southeast Asia
results_diab_sea <- run_emm_analysis(data = mydata, outcome_var = "diabetes", region = "SEARD")
results_diab_sea $sex_summary
results_diab_sea $age_summary
results_diab_sea $edu_summary

# Diabetes Western Pacific
results_diab_wp <- run_emm_analysis(data = mydata, outcome_var = "diabetes", region = "Western Pacific")
results_diab_wp $sex_summary
results_diab_wp $age_summary
results_diab_wp $edu_summary

#################################################################
####-------------Interaction results for Hypertesion----------##
################################################################
# Hypertesion global
results_diab_global <- run_emm_analysis(data = mydata, outcome_var = "htn")
results_diab_global$sex_summary
results_diab_global$age_summary
results_diab_global$edu_summary

# Hypertesion Africa
```

© 2025 Ahmed KY et al. *JAMA Network Open*.

```
results_diab_africa <- run_emm_analysis(data = mydata, outcome_var = "htn", region = "Africa")

results_diab_africa$sex_summary

results_diab_africa$age_summary

results_diab_africa$edu_summary


# Hypertesion Americas

results_diab_americas <- run_emm_analysis(data = mydata, outcome_var = "htn", region = "Americas")

results_diab_americas$sex_summary

results_diab_americas$age_summary

results_diab_americas$edu_summary


# Hypertesion Eastern Mediterranean

results_diab_em <- run_emm_analysis(data = mydata, outcome_var = "htn", region = "Eastern Mediterranean")

results_diab_em $sex_summary

results_diab_em $age_summary

results_diab_em $edu_summary


# Hypertesion Europe

results_diab_eu <- run_emm_analysis(data = mydata, outcome_var = "htn", region = "Europe")

results_diab_eu $sex_summary

results_diab_eu $age_summary

results_diab_eu $edu_summary


# Hypertesion Southeast Asia

results_diab_sea <- run_emm_analysis(data = mydata, outcome_var = "htn", region = "SEARD")

results_diab_sea $sex_summary

results_diab_sea $age_summary

results_diab_sea $edu_summary


# Hypertesion Western Pacific

results_diab_wp <- run_emm_analysis(data = mydata, outcome_var = "htn", region = "Western Pacific")

results_diab_wp $sex_summary

results_diab_wp $age_summary

results_diab_wp $edu_summary
```

© 2025 Ahmed KY et al. *JAMA Network Open*.

```
################################################################

####-------------Interaction results for Triglycerides----------##

###############################################################


# Triglycerides global

results_diab_global <- run_emm_analysis(data = mydata, outcome_var = "Triglycerides_binary")

results_diab_global$sex_summary

results_diab_global$age_summary

results_diab_global$edu_summary



# Triglycerides Africa

results_diab_africa <- run_emm_analysis(data = mydata, outcome_var = "Triglycerides_binary", region = "Africa")

results_diab_africa$sex_summary

results_diab_africa$age_summary

results_diab_africa$edu_summary


# Triglycerides Americas

results_diab_americas <- run_emm_analysis(data = mydata, outcome_var = "Triglycerides_binary", region = "Americas")

results_diab_americas$sex_summary

results_diab_americas$age_summary

results_diab_americas$edu_summary


# Triglycerides Eastern Mediterranean

results_diab_em <- run_emm_analysis(data = mydata, outcome_var = "Triglycerides_binary", region = "Eastern
Mediterranean")

results_diab_em $sex_summary

results_diab_em $age_summary

results_diab_em $edu_summary


# Triglycerides Europe

results_diab_eu <- run_emm_analysis(data = mydata, outcome_var = "Triglycerides_binary", region = "Europe")

results_diab_eu $sex_summary

results_diab_eu $age_summary
```

© 2025 Ahmed KY et al. *JAMA Network Open*.

```
results_diab_eu $edu_summary

# Triglycerides Southeast Asia
results_diab_sea <- run_emm_analysis(data = mydata, outcome_var = "Triglycerides_binary", region = "SEARD")
results_diab_sea $sex_summary
results_diab_sea $age_summary
results_diab_sea $edu_summary

# Triglycerides Western Pacific
results_diab_wp <- run_emm_analysis(data = mydata, outcome_var = "Triglycerides_binary", region = "Western Pacific")
results_diab_wp $sex_summary
results_diab_wp $age_summary
results_diab_wp $edu_summary

################################################################
####-------------Interaction results for Cholesterol----------##
###############################################################
# Cholesterol global
results_diab_global <- run_emm_analysis(data = mydata, outcome_var = "cholestrol")
results_diab_global$sex_summary
results_diab_global$age_summary
results_diab_global$edu_summary

# Cholesterol Africa
results_diab_africa <- run_emm_analysis(data = mydata, outcome_var = "cholestrol", region = "Africa")
results_diab_africa$sex_summary
results_diab_africa$age_summary
results_diab_africa$edu_summary

# Cholesterol Americas
results_diab_americas <- run_emm_analysis(data = mydata, outcome_var = "cholestrol", region = "Americas")
results_diab_americas$sex_summary
results_diab_americas$age_summary
results_diab_americas$edu_summary
```

© 2025 Ahmed KY et al. *JAMA Network Open*.

```
# Cholesterol Eastern Mediterranean
results_diab_em <- run_emm_analysis(data = mydata, outcome_var = "cholestrol", region = "Eastern Mediterranean")
results_diab_em $sex_summary
results_diab_em $age_summary
results_diab_em $edu_summary

# Cholesterol Europe
results_diab_eu <- run_emm_analysis(data = mydata, outcome_var = "cholestrol", region = "Europe")
results_diab_eu $sex_summary
results_diab_eu $age_summary
results_diab_eu $edu_summary

# Cholesterol Southeast Asia
results_diab_sea <- run_emm_analysis(data = mydata, outcome_var = "cholestrol", region = "SEARD")
results_diab_sea $sex_summary
results_diab_sea $age_summary
results_diab_sea $edu_summary

# Cholesterol Western Pacific
results_diab_wp <- run_emm_analysis(data = mydata, outcome_var = "cholestrol", region = "Western Pacific")
results_diab_wp $sex_summary
results_diab_wp $age_summary
results_diab_wp $edu_summary
```

© 2025 Ahmed KY et al. *JAMA Network Open*.
