# Supplementary material for: Cardiometabolic Outcomes Among Adults With Abdominal Obesity and Normal Body Mass Index
Source: JAMA Netw Open. 2025 Oct 17;8(10):e2537942. doi: 10.1001/jamanetworkopen.2025.37942 (PMC12534855; doi:10.1001/jamanetworkopen.2025.37942)
Supplement: Supplement 2. — Data Sharing Statement [file jamanetwopen-e2537942-s002.pdf]

Data Sharing Statement

Ahmed. Cardiometabolic Outcomes Among Adults With Abdominal Obesity and Normal Body Mass Index. *JAMA Netw Open*. Published October 17, 2025.
doi:10.1001/jamanetworkopen.2025.37942

Data
Data available: No

Additional Information
Explanation for why data not available: This study is a secondary analysis of WHO STEPS survey datasets. Information on the data and content can be accessed at https://extranet.who.int/ncdsmicrodata/index.php/home.
